# Structural Evolution of a Crustal‐Scale Seismogenic Fault in a Magmatic Arc: The Bolfin Fault Zone (Atacama Fault System)

**DOI:** 10.1029/2021TC006818

**Published:** 2021-08-09

**Authors:** Simone Masoch, Rodrigo Gomila, Michele Fondriest, Erik Jensen, Thomas Mitchell, Giorgio Pennacchioni, José Cembrano, Giulio Di Toro

**Affiliations:** ^1^ Dipartimento di Geoscienze Università degli Studi di Padova Padua Italy; ^2^ Institut des Sciences de la Terre (ISTerre) Université Grenoble‐Alpes Grenoble France; ^3^ CIGIDEN Santiago Chile; ^4^ Department of Earth Sciences University College London London UK; ^5^ Departamento de Ingeniería Estructural y Geotécnica Pontificia Universidad Católica de Chile Santiago Chile; ^6^ Andean Geothermal Center of Excellence (CEGA, FONDAP‐CONICYT) Santiago Chile; ^7^ Sezione di Tettonofisica e Sismologia Istituto Nazionale di Geofisica e Vulcanologia Rome Italy

**Keywords:** seismogenic fault, fault growth, structural inheritance, pseudotachylytes, intra‐arc deformation, Atacama Fault System

## Abstract

How major crustal‐scale seismogenic faults nucleate and evolve in crystalline basements represents a long‐standing, but poorly understood, issue in structural geology and fault mechanics. Here, we address the spatio‐temporal evolution of the Bolfin Fault Zone (BFZ), a >40‐km‐long exhumed seismogenic splay fault of the 1000‐km‐long strike‐slip Atacama Fault System. The BFZ has a sinuous fault trace across the Mesozoic magmatic arc of the Coastal Cordillera (Northern Chile) and formed during the oblique subduction of the Aluk plate beneath the South American plate. Seismic faulting occurred at 5–7 km depth and ≤ 300°C in a fluid‐rich environment as recorded by extensive propylitic alteration and epidote‐chlorite veining. Ancient (125–118 Ma) seismicity is attested by the widespread occurrence of pseudotachylytes. Field geologic surveys indicate nucleation of the BFZ on precursory geometrical anisotropies represented by magmatic foliation of plutons (northern and central segments) and andesitic dyke swarms (southern segment) within the heterogeneous crystalline basement. Seismic faulting exploited the segments of precursory anisotropies that were optimal to favorably oriented with respect to the long‐term far‐stress field associated with the oblique ancient subduction. The large‐scale sinuous geometry of the BFZ resulted from the hard linkage of these anisotropy‐pinned segments during fault growth.

## Key Points

- We describe the structural evolution of the Bolfin Fault Zone, a >40‐km‐long exhumed seismogenic fault of the Atacama Fault System
- Seismic faulting exploited magmatic foliations and dykes well‐oriented with respect to the subduction‐related stress field
- The sinuous geometry of the Bolfin Fault Zone results from hard linkage of anisotropy‐pinned fault segments during fault growth

## 1 Introduction

Most continental crustal deformation is localized into ductile shear zones and brittle, commonly seismogenic, faults (e.g., Snoke et al., 1998). The nucleation and evolution of brittle faults in the upper continental crust is associated with (i) formation of new fractures whose orientation is controlled by the regional or local stress field based on rock failure criteria (e.g., Anderson, 1951; Chemenda et al., 2016; Jaeger et al., 2009; Mandl, 1988; Naylor et al., 1986; Pennacchioni & Mancktelow, 2013; Swanson, 1999a, 1999b; 2006a; Woodcock, 1986), or (ii) exploitation of pre‐existing structures (e.g., fractures, bedding, stratigraphic contacts, fold hinges and limbs, dykes, ductile shear zones, etc.: Crider, 2015; Crider & Peacock, 2004; d'Alessio & Martel, 2005; Davatzes & Aydin, 2003; Fondriest et al., 2020, 2012; Mandl, 1988; Martel, 1990; Mittempergher et al., 2021; Nasseri et al., 2003, 1997; Pachell & Evans, 2002; Peacock & Sanderson, 1995; Pennacchioni et al., 2006; Segall & Pollard, 1983; Sibson, 1990; Smith et al., 2013; Swanson, 1988, 2006b; Sylvester, 1988). Geologic and geophysical studies, rock analog experiments, and numerical models have highlighted that crustal‐scale (i.e., tens of km‐long) brittle faults commonly exploit exhumed mylonitic horizons in the crystalline basement (e.g., Balázs et al., 2018; Bellahsen & Daniel, 2005; Bistacchi et al., 2010, 2012; Butler et al., 2008; Collanega et al., 2019; Hodge et al., 2018; Holdsworth et al., 2011; Massironi et al., 2011; Naliboff et al., 2020; Phillips et al., 2019; Stewart et al., 2000; Storti et al., 2003; Sylvester, 1988; Wedmore et al., 2020; Whipp et al., 2014). Nevertheless, only a few contributions have attempted to evaluate the influence of precursory structures over crustal‐scale faults (e.g., Bistacchi et al., 2010; Hodge et al., 2018; Wedmore et al., 2020). In particular, the influence of magmatic‐related structures, such as magmatic foliation of plutons and dyke swarms, on fault nucleation and segmentation is poorly investigated. Moreover, the evolution in space and time of crustal‐scale, *seismogenic* faults remains poorly known (e.g., Kirkpatrick et al., 2013; Perrin et al., 2016; Shigematsu et al., 2017; Williams et al., 2017). Indeed, moderate to large in magnitude (*M* > 6) earthquakes rupture faults extending for >15 km in length, but such large faults are rarely well‐exposed along their whole length at the surface due to weathering and vegetation or Quaternary cover. Major mature faults typically record a long, polyphase deformation history, which might obliterate the incipient stages of nucleation and growth (e.g., Rizza et al., 2019). Thus, the field geologists' challenge in studying ancient, crustal‐scale seismogenic fault systems is to find large areas which meet the following criteria:

- excellent preservation over kilometer‐scale exposures of the spatial arrangement of structures (e.g., joints, dykes, and faults) related to multiple deformation stages;
- faults exhumed from depths (i.e., 5–15 km depending on tectonic regime, rock composition, temperature gradient, etc.; Scholz, 2019), where moderate to large in magnitude earthquakes nucleate in the continental crust
- presence of tectonic pseudotachylytes (i.e., solidified frictional melts), unambiguous evidence of seismic slip in the rock record (Cowan, 1999; Rowe & Griffith, 2015; Sibson, 1975).

**Figure 1:**
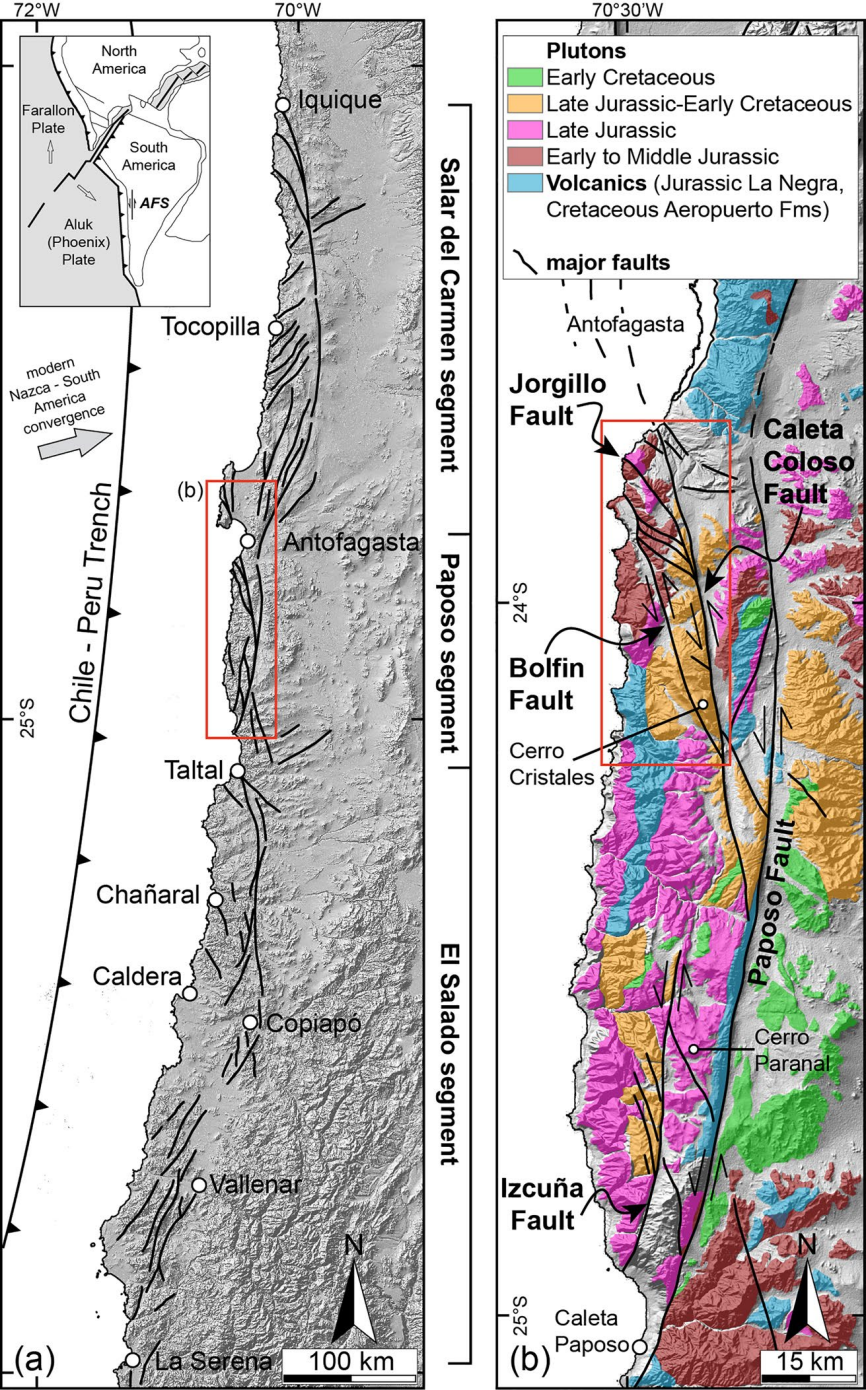
Tectonic setting of the Atacama Fault System (AFS) in the Coastal Cordillera (Atacama Desert, Northern Chile). (a) Crustal‐scale geometry of the AFS with its three concave‐shaped main segments. Shaded‐relief image modified from Cembrano et al. (2005) and Veloso et al. (2015). Red box indicates the area shown in (b). The inset map shows the approximate plate configuration coeval with the Mesozoic sinistral strike‐slip deformation along the AFS. Redrawn from Jaillard et al. (1990). (b) Simplified geologic map of the Coastal Cordillera along the Paposo segment. Igneous lithologies are mapped with color coding by age. Unmapped areas represent metamorphic units and sedimentary covers. Data compiled and simplified from Cembrano et al. (2005), Domagala et al. (2016), González and Niemeyer (2005), and SERNAGEOMIN (2003). DTM base layer elaborated from USGS Aster GDEM database (https://earthexplorer.usgs.gov/) as base map. Red box indicates the studied area (Figures 2 and 3).

**Figure 2:**
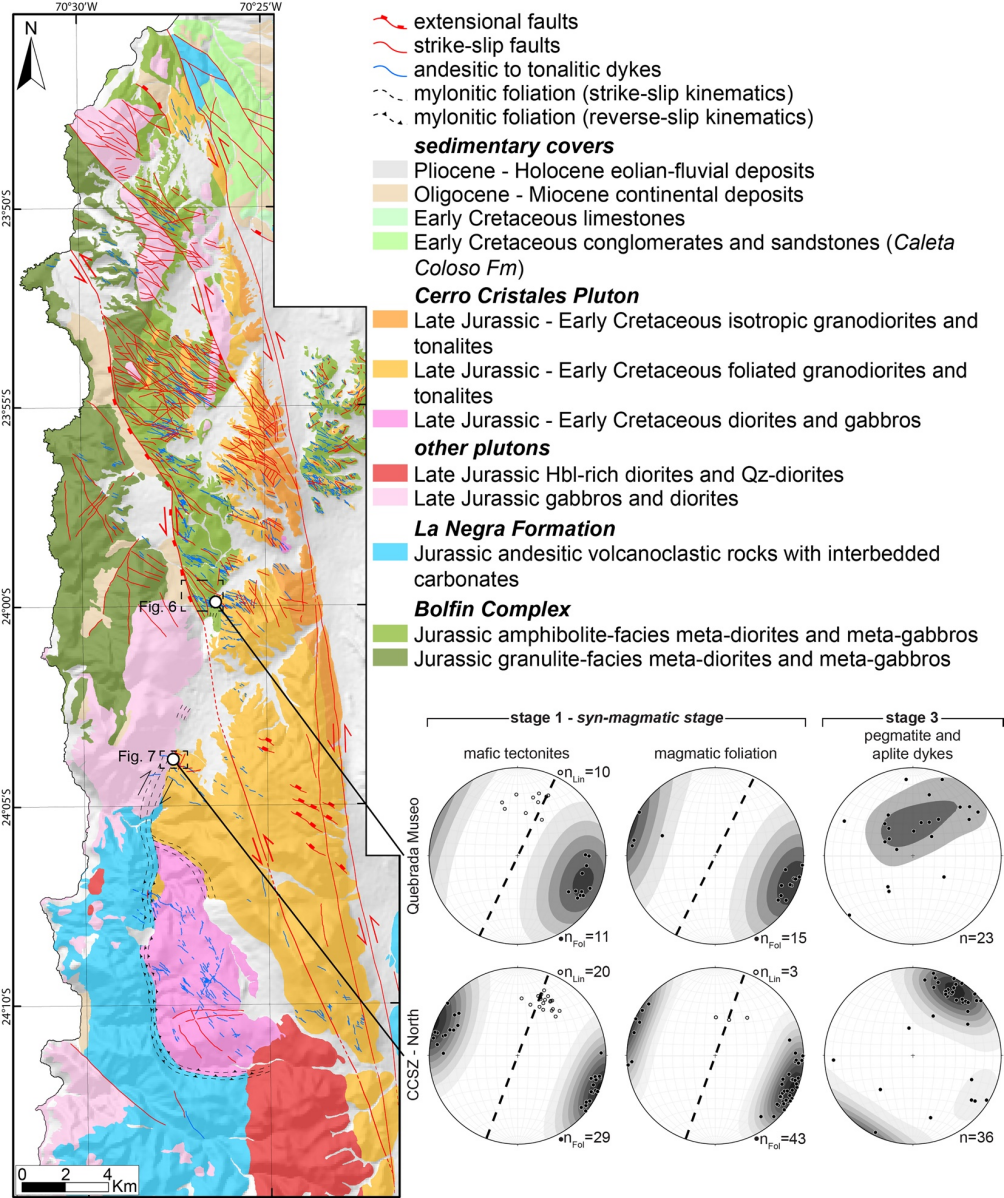
Geologic map of the Bolfin area and structural data from the two localities along the Cerro Cristales Shear Zone (CCSZ). Stereoplots (lower hemisphere, equal area) display (i) poles‐to‐planes (bold circles) of high‐temperature foliation, magmatic foliation, and aplite/pegmatite dykes, and (ii) mineral stretching lineations (open circles) within high‐temperature and magmatic foliations. In the stereoplots, the dashed line indicates the mean attitude of the CCSZ at each locality. The structural data of deformation stage 2, associated with the ductile reworking of dykes, are presented in Figure 5c. Dashed black boxes indicate the locations of geologic maps in Figures 6 and 7.

The sinistral strike‐slip Atacama Fault System (AFS) in the Coastal Cordillera (Northern Chile; Figure 1) (Arabasz, 1971; Cembrano et al., 2005; Scheuber & González, 1999), associated with the ancient subduction of the Aluk (Phoenix) plate beneath the South America plate, is well exposed along strike over more than 1,000 km. The exceptional outcrop conditions result from the hyper‐arid climate since 25–22 Ma (Dunai et al., 2005) and the slow erosion rates in the Atacama Desert. This makes the AFS an outstanding setting for studying the structural evolution of major faults hosted in the continental crust. Here, we consider the Middle‐Late Jurassic‐Early Cretaceous sequence of magmatic, solid‐state, and brittle deformation that developed along the Northern Paposo segment of the AFS. Specifically, we consider the evolution of the >40‐km‐long seismogenic Bolfin Fault Zone (BFZ) and the large‐scale syn‐magmatic to post‐magmatic Cerro Cristales Shear Zone (CCSZ) (Figures 1 and 2). The study integrates (a) geologic field mapping, (b) analysis of satellite and drone images, and (c) microstructural investigations of fault zone rocks and host rocks. We show that the large‐scale sinuous geometry of the seismogenic BFZ is imposed by the local pinning of fault orientation on magmatic structures related to the precursory history of the magmatic arc. The BFZ was seismogenic, as attested by widespread occurrence of pseudotachylytes, and active at ambient temperatures of ≤ 300°C and depths of 5–7 km in a fluid‐rich environment. We conclude that magmatic‐related structures, such as foliated plutons and dyke swarms, may partly control the nucleation, evolution, and geometry of crustal‐scale seismogenic faults.

## 2 Geologic Setting

### 2.1 Coastal Cordillera

The Coastal Cordillera represents the Jurassic‐Early Cretaceous continental magmatic arc, formed during oblique subduction of the Aluk (Phoenix) oceanic plate underneath the South America plate (inset in Figure 1a) (Parada et al., 2007, and references therein). The magmatic arc is mainly composed of gabbro to granodiorite plutons and basaltic to andesitic volcanic rocks (La Negra Formation) (Figure 1b). Mesozoic plutons are large, elongated (N‐S) bodies intruded at middle to upper crustal levels within a Paleozoic metamorphic basement (Mejillones Metamorphic Complex and Chañaral Mélange) (Hervé et al., 2007, and references therein).

Since Middle‐Late Jurassic, growth of the magmatic arc was associated with nearly arc‐perpendicular crustal extension, under a Mariana‐type subduction. This promoted the emplacement of large intrusions at shallow depth (Brown et al., 1993; Grocott et al., 1994; Grocott & Taylor, 2002; Scheuber & González, 1999). This stage is recorded by N‐S trending extensional brittle faults and ductile shear zones, and homoclinal tilting of Jurassic La Negra Formation (Brown et al., 1993; Scheuber & González, 1999). During Late Jurassic‐Early Cretaceous, intra‐arc dextral transtension induced the emplacement of NE‐striking andesitic dykes (Scheuber & González, 1999). Since the Early Cretaceous, the Coastal Cordillera underwent intra‐arc sinistral and sinistral transtensional deformation due to the SE‐directed oblique subduction (Scheuber & González, 1999). This deformation stage is recorded by the emplacement of NW‐striking andesitic dykes and the formation of the trench‐parallel AFS (Scheuber & Andriessen, 1990; Scheuber & González, 1999).

### 2.2 Atacama Fault System

The 1000‐km‐long AFS is the major crustal‐scale, strike‐slip fault system within the present‐day forearc of the Central Andes (Figure 1) (Arabasz, 1971; Brown et al., 1993; Cembrano et al., 2005; Scheuber & González, 1999). The AFS includes three main, curved segments (from north to south): (i) Salar del Carmen, (ii) Paposo, and (iii) El Salado (Figure 1a).

The AFS developed in the Early Cretaceous accommodating intra‐arc sinistral and sinistral transtensional deformation, once the axis of arc magmatism migrated eastwards (Scheuber & González, 1999; Scheuber et al., 1995). Brittle faults overprinted mylonites of similar kinematics and the age of ductile and brittle deformation varies along strike (Brown et al., 1993; Scheuber & González, 1999; Scheuber et al., 1995; Seymour et al., 2020, 2021). Along the Paposo segment, in the outer shell of the Cerro Paranal Pluton, syn‐mylonitic hornblende and biotite yield ^40^Ar/^39^Ar and Rb‐Sr ages in the range between 138 and 125 Ma (Scheuber et al., 1995). Similar zircon U‐Pb ages, referred to the onset of ductile deformation, were reported in the southern Paposo segment (∼139 Ma; Ruthven et al., 2020). Brittle faulting was constrained between 125 and 118 Ma (Olivares et al., 2010; Scheuber & Andriessen, 1990). Extensional faulting along the AFS was reported during Miocene to post‐Miocene in response to large magnitude subduction earthquakes (e.g., González et al., 2003, 2006).

### 2.3 Geology of the Bolfin Area (23°45′S–24°15′S)

**Figure 3:**
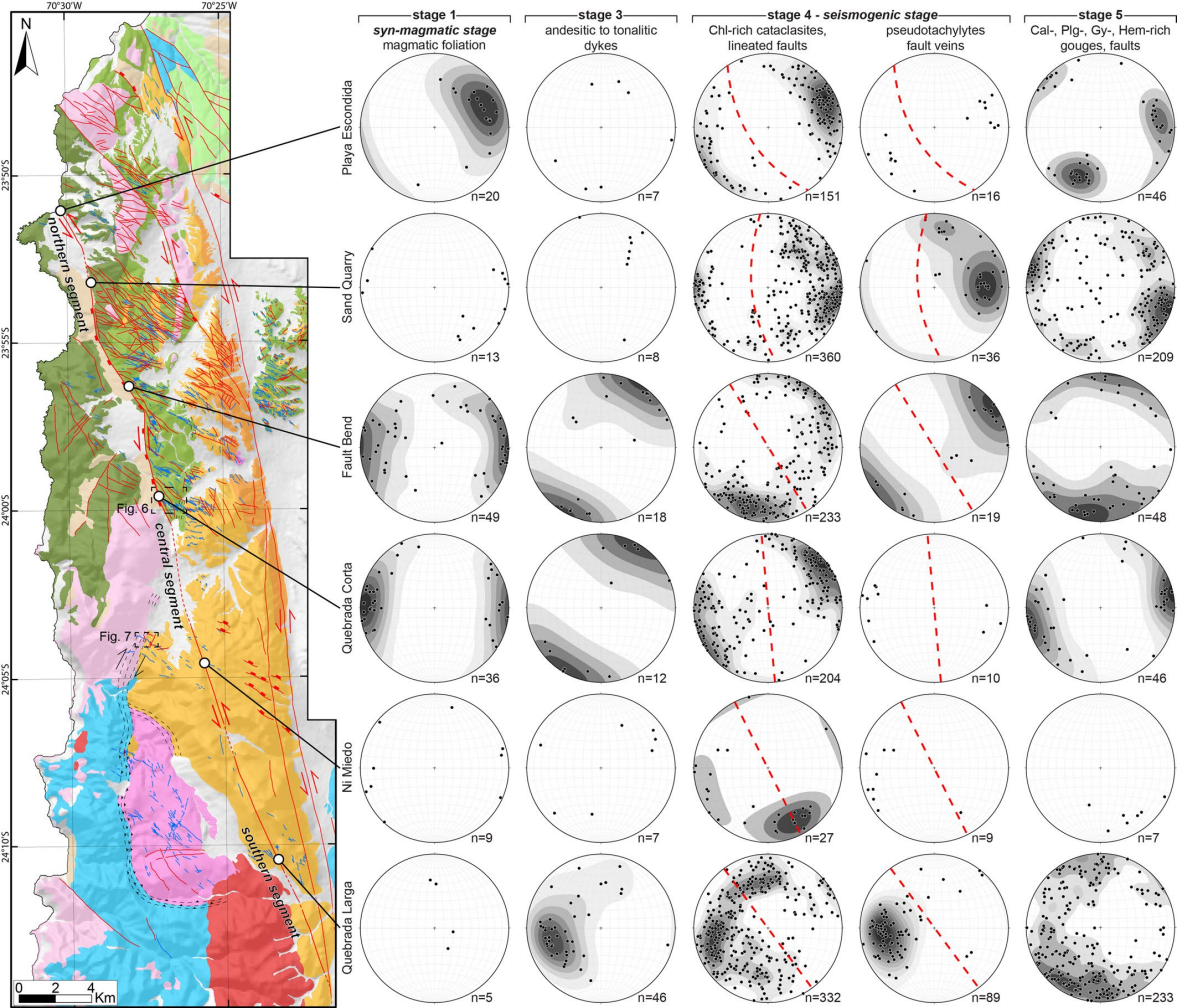
Geologic map of the Bolfin area and structural data from the six localities along the Bolfin Fault Zone (BFZ) divided by deformation stages (see Figure 2 for the map legend). In the stereoplots (lower hemisphere, equal area), the dashed red line indicates the mean attitude of the fault core of the BFZ. Structural data of deformation stage 2, associated with the ductile reworking of dykes, are presented in Figure 5c. Dashed black boxes indicate the locations of geologic maps in Figures 6 and 7.

The crystalline basement of the Bolfin area consists of (a) Early Middle Jurassic meta‐igneous Bolfin Complex, (b) Late Jurassic plutons and (c) Late Jurassic to Early Cretaceous Cerro Cristales Pluton, and (d) volcanoclastic rocks of the La Negra Formation (Figures 1, 2, 3). The Bolfin Complex consists of diorites and gabbros, partially to completely recrystallized at amphibolite‐granulite facies, metamorphic conditions (González & Niemeyer, 2005; Lucassen & Franz, 1994; Lucassen & Thirlwall, 1998).

The Cerro Cristales Pluton, formed by tonalite‐granodiorite and diorite‐quartz‐diorite units (Domagala et al., 2016; González, 1999; González & Niemeyer, 2005), is an NNE‐elongated body showing an outer shell of strongly foliated rocks (Figures 2 and 3). The eastern and southern contact between the pluton and host rocks is marked by the large‐scale CCSZ (González, 1999) (Figure 2). According to González (1999), the CCSZ is a sinistral strike‐slip ductile shear zone active at amphibolite‐facies conditions, favoring and controlling the emplacement of the pluton. NW‐striking syn‐kinematic diorite and andesite dykes cut both the plutonic and volcanic rocks. These dykes represent the last magmatic event coeval with the formation of the AFS (Olivares et al., 2010; Scheuber & González, 1999).

Large‐scale, sinistral strike‐slip faults of the AFS cut through the crystalline rocks (Figures 1, 2, 3). Some of the N‐striking major faults and NW‐striking to NNW‐striking splay faults are hierarchically organized into strike‐slip duplexes over a wide range of scales (Cembrano et al., 2005; Jensen et al., 2011; Veloso et al., 2015). Most of the splay faults accommodated displacements up to a few kilometers (Cembrano et al., 2005; Gomila et al., 2016; Mitchell & Faulkner, 2009). Brittle faulting occurred at 5–7 km depth at greenschist‐facies to sub‐greenschist‐facies conditions (280–350°C: Arancibia et al., 2014; Cembrano et al., 2005). Faulting developed chlorite‐rich cataclasites, associated with pervasive syn‐kinematic hydrothermal activity attested by the widespread occurrence of epidote‐rich and chlorite‐rich faults and veins (Arancibia et al., 2014; Cembrano et al., 2005; Herrera et al., 2005; Olivares et al., 2010). In our study, we focus on chlorite‐rich cataclastic rocks.

The BFZ is a third‐order fault of the AFS bounding the kilometer‐scale Caleta Coloso Duplex (Figures 1, 2, 3) (Cembrano et al., 2005; Herrera et al., 2005; Olivares et al., 2010). The Early Cretaceous strike‐slip structure of the BFZ was overprinted by Late Cenozoic extensional faulting. During this later stage, Miocene‐to‐Pliocene continental deposits were juxtaposed with chlorite‐rich cataclasites of the BFZ fault core.

## 3 Methods

Original field structural surveys along with remote sensing analysis were performed to characterize the regional‐scale pattern of tectonic lineaments (i.e., faults and shear zones) and dykes in the study area (20‐km wide, 50‐km long). Remote sensing analysis was performed using satellite images (i.e., Sentinel‐2, Google Earth and Bing) as reference maps coupled with published geologic and structural maps (Cembrano et al., 2005; Domagala et al., 2016; González & Niemeyer, 2005). Six representative localities along the BFZ and two along the CCSZ were selected for a detailed analysis (Figures 2 and 3). At each locality, we used a DJI Phantom 4 Pro drone to take nadir‐directed aereophotographs. The images were processed in Agisoft Metashape Professional software to generate high‐resolution georeferenced orthomosaics (spatial resolution of ∼10 cm/pixel) used as base maps for the surveys at 1:300, 1:500, or 1:1,000 scale. The orientation and kinematics of the different structural elements (magmatic foliations, dykes, joints, faults, and ductile shear zones) were systematically measured and digitalized using ArcGIS 10.6 software. Structural measurements (*n* = 2,716) were plotted onto stereonets (equal area, low hemisphere) using Stereonet 10 (Allmendinger et al., 2011; Cardozo & Allmendinger, 2013).

Oriented rock samples (*n* = 178) were collected for microanalytical investigations. Microstructural observations were conducted on polished thin sections (*n* = 60) oriented parallel to the *X* kinematic direction (stretching lineation and slickenline in shear zones and faults, respectively) and orthogonal to the *X*‐*Y* plane (shear zone boundary and fault plane). Transmitted‐light optical microscopy (OM) was used to determine microstructural features at thin‐section scale and to identify areas suitable for microanalytical investigations. Scanning electron microscopy (SEM) was used to acquire high‐resolution backscattered electron (BSE) images coupled with semiquantitative energy dispersion spectroscopy (EDS) elemental analysis. SEM and field‐emission SEM investigations were performed with a CamScan MX3000 operating at 25 kV at the Department of Geosciences at Università Degli Studi di Padova and a JOEL JSM‐6500F operating at 15 kV at HP‐HT laboratories of Istituto Nazionale di Geofisica e Vulcanologia (INGV) in Rome, respectively. Bulk mineralogy of rock samples was retrieved through X‐ray powder diffraction (XRPD), and semiquantitative mineralogical composition was retrieved through Reference Intensity Ratio (XRPD‐RIR) method. XRPD analyses were performed with a PANalytical X'Pert Pro diffractometer equipped with a Co radiation source, operating at 40 mA and 40 kV in the angular range 3° < 2*θ* < 85°, installed at the Department of Geosciences (Padova). Mineral composition of main mineral phases was obtained by electron wavelength‐dispersive microprobe analysis (EMPA). EMPA investigations were performed with a Joel‐JXA8200 microprobe equipped with EDS‐WDS (5 spectrometers with 12 crystals), installed at INGV‐Rome, and a Cameca SX50 microprobe, installed at the Department of Geosciences (Padova). Data were collected using 15 kV as accelerating voltage and 7.5 nA as beam current. A slightly defocused electron beam with a size of 5 μm was used, with a counting time of 5 s on background and 10 s on peak. Albite (Si, Al, and Na), forsterite (Mg), pyrite (Fe), rutile (Ti), orthoclase (K), and apatite (Ca and P) were used as standards. Sodium and potassium were analyzed first to prevent alkali migration effects. The precision of the microprobes was measured through the analysis of well‐characterized synthetic oxide and mineral secondary standards. Based on counting statistics, analytical uncertainties relative to their reported concentrations indicate that precision was better than 5% for all cations.

## 4 Field Observations

We describe the spatial distribution and attitude data for magmatic, solid‐state, and brittle deformation structures for eight localities. Two localities are along the CCSZ, at the contact between the Cerro Cristales Pluton and either the meta‐diorite of the Bolfin Complex (Quebrada Museo) or the Late Jurassic gabbro of the Cerro Mulato (CCSZ‐North) (see locations in Figure 2). Six localities are along the BFZ and are referred to as Playa Escondida, Sand Quarry, Fault Bend, Quebrada Corta (within the Bolfin Complex), Ni Miedo, and Quebrada Larga (within the Cerro Cristales tonalite‐granodiorite) (see locations in Figure 3).

### 4.1 Dyke Generations

**Figure 4:**
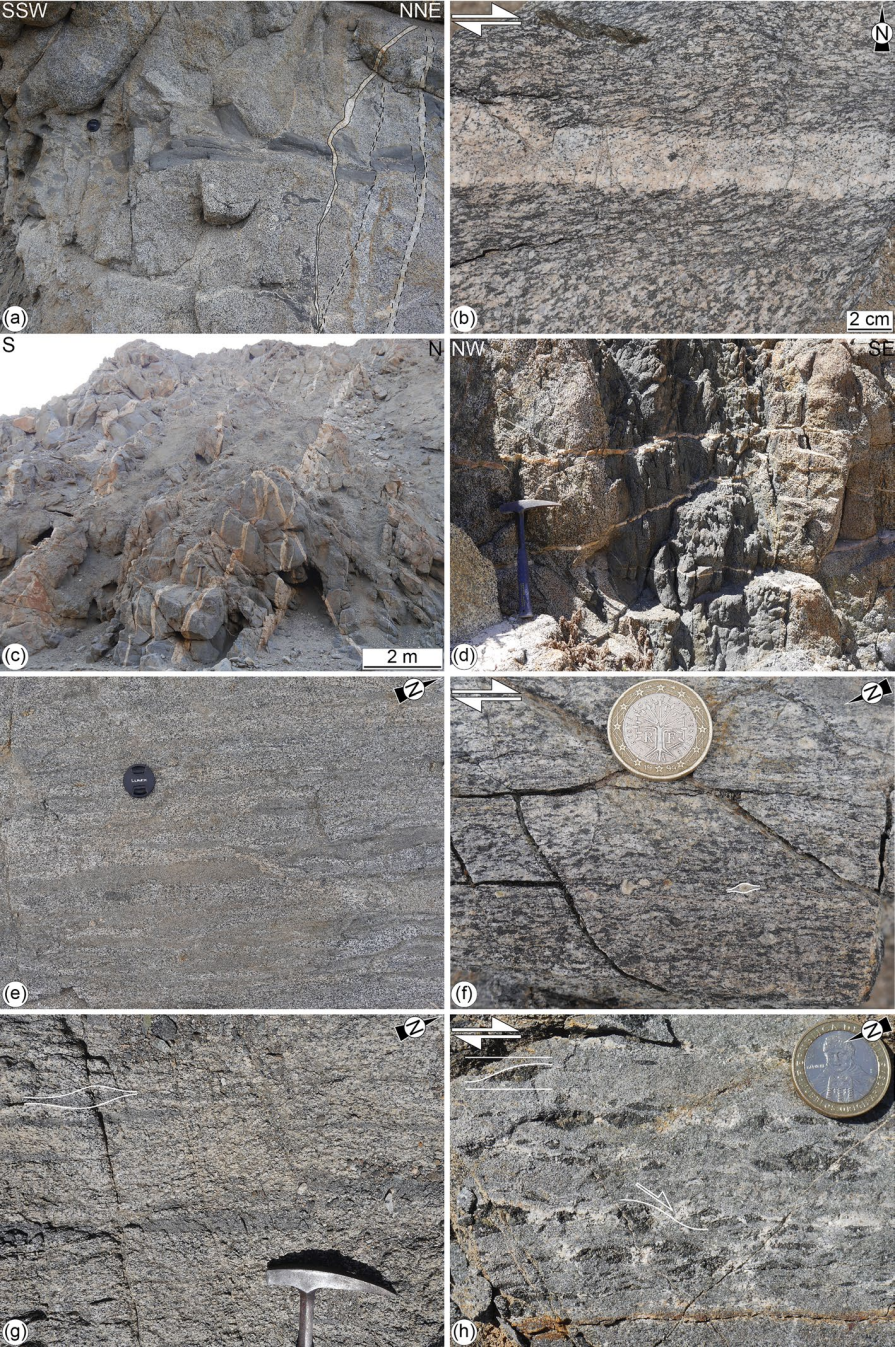
Dykes and syn‐magmatic to post‐magmatic high‐temperature structures related to the tectono‐magmatic evolution of the studied area (**deformation stages 1–3** or pre‐Bolfin Fault Zone s.s.). (a) Dismembered amphibolitic dyke (**deformation stage 1**) within the foliated meta‐diorites of the Bolfin Complex. The black amphibolitic dyke is cut by leucocratic (dashed black lines; **deformation stage 2**) and pegmatite dykes (solid lines; **deformation stage 3**). Lens cover for scale; cover width: 5.2 cm. WGS84 GPS location: 24.000489°S, 70.442743°W. (b) Paired ductile shear zone at the boundary of a leucocratic dyke within the meta‐diorites of the Bolfin Complex (**deformation stage 2**). Dextral sense of shear. WGS84 GPS location: 23.999886°S, 70.444403°W. (c) Pink pegmatite dykes cutting the Cerro Cristales Shear Zone at CCSZ‐North locality (**deformation stage 3**). WGS84 GPS location: 24.063805°S, 70.457332°W. (d) Shallow dipping aplite dykes (pink; **deformation stage 3**) cutting a dark gray amphibolitic dyke (**deformation stage 1**). Hammer for scale; height 33 cm. WGS84 GPS location: 24.000409°S, 70.443231°W. (e) Foliated meta‐diorite of the Bolfin Complex close to the contact with the CCSZ at Quebrada Museo locality (**deformation stage 1**). Mafic enclaves define the magmatic foliation. Lens cover for scale. WGS84 GPS location: 23.9983694°S, 70.4409305°W. (f) Mafic tectonite of the CCSZ (**deformation stage 1**, Quebrada Museo locality). Sigmoidal amphiboles and sigma‐type plagioclase porphyroclasts (white lines) indicate dextral sense of shear. XZ section. 1‐euro coin for scale. WGS84 GPS location: 23.9967600°S, 70.4396600°W. (g) Foliated tonalite of the Cerro Cristales Pluton (**deformation stage 1**, CCSZ‐North locality). The magmatic foliation is defined by asymmetric mafic microgranular enclaves (white lines) indicating dextral kinematics. Hammer for scale; head width: 18 cm. WGS84 GPS location: 24.062278°S, 70.456497°W. (h) Mafic tectonite along the CCSZ at CCSZ‐North (**deformation stage 1**). Sigmoidal amphibole lenses and shear band boudins indicate dextral sense of shear. Nearly XZ section. 100‐pesos coin for scale. WGS84 GPS location: 24.062922°S, 70.460754°W.

**Figure 5:**
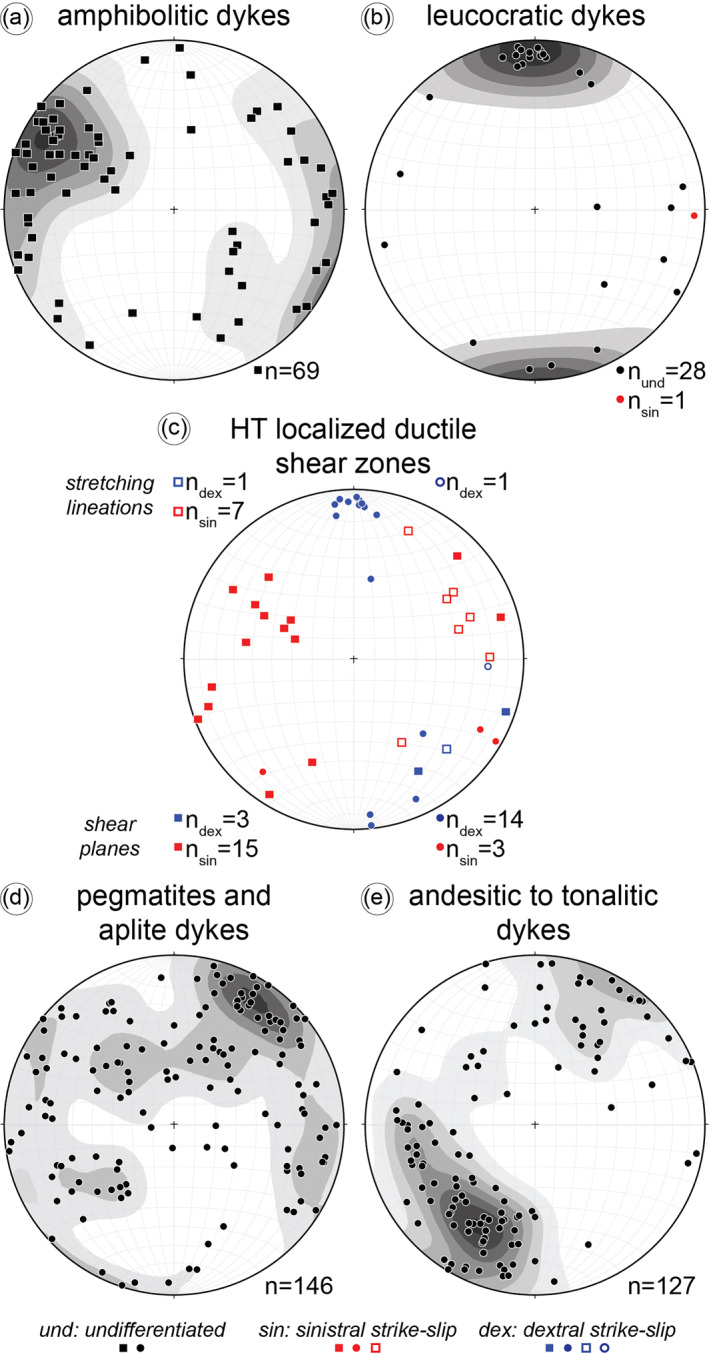
Lower hemisphere, equal area stereonets of poles to (a) amphibolitc dykes, (b) leucocratic dykes, (c) small‐scale high‐temperature ductile shear zones (associated with the **deformation stage 2**) exploiting the amphibolitic dykes (squares) and nucleating at the external boundaries of the leucocratic dykes (circles), (d) pegmatite and aplite dykes (cumulative stereographic plot) and (e) andesitic to tonalitic dykes (cumulative stereographic plot). The structural data are from all the eight studied localities along the Cerro Cristales Shear Zone (Figure 2) and the Bolfin Fault zone (Figure 3). Solid symbols indicate planes and shear planes, whereas open ones indicate mineral stretching lineations.

**Figure 6:**
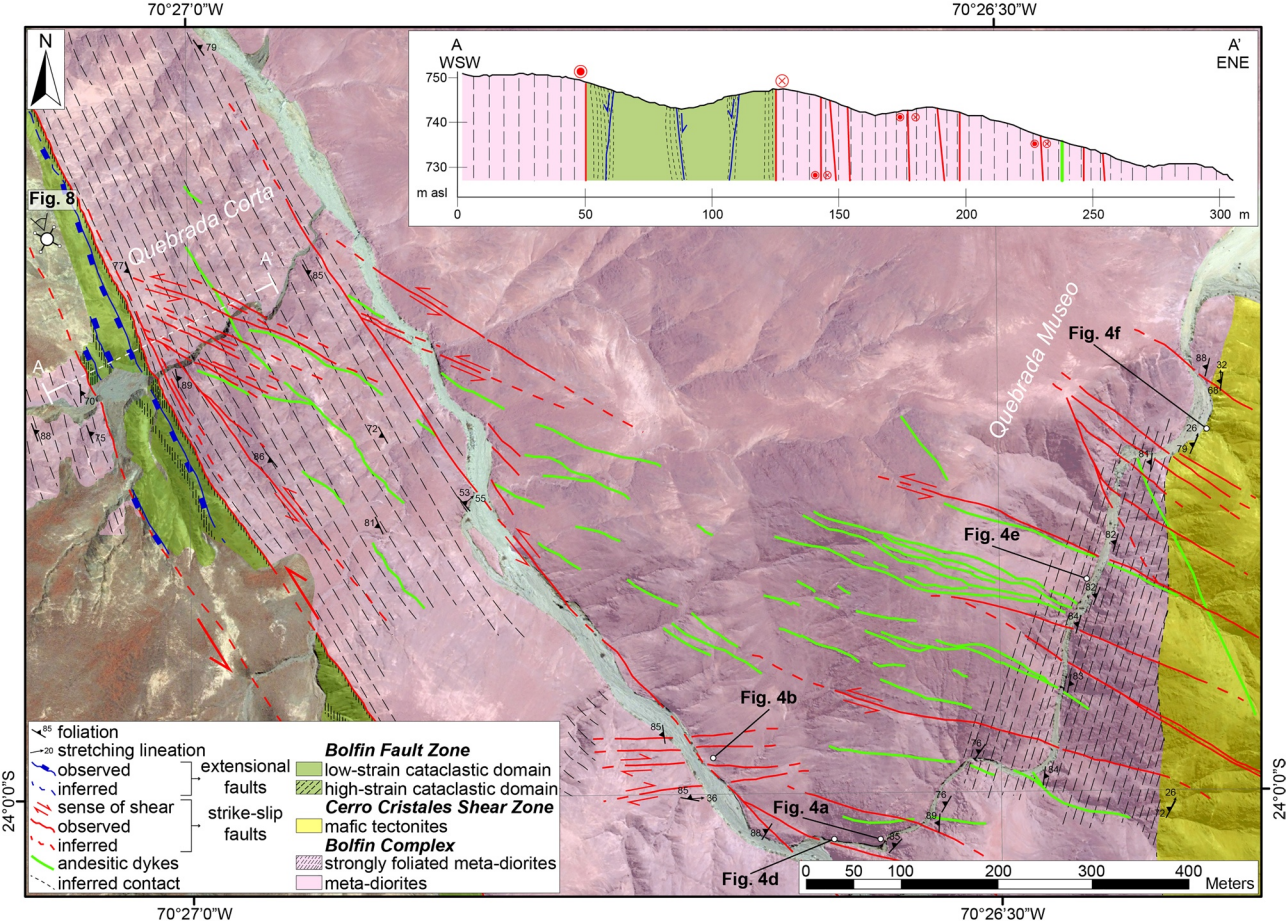
Detailed geologic map of Quebrada Corta and Quebrada Museo localities along the Bolfin Fault Zone (BFZ) and the Cerro Cristales Shear Zone, respectively, and geologic cross‐section across the BFZ. The cross‐section is oriented N64°E (i.e., perpendicular to the fault core strike). The ∼75‐m‐wide cataclastic fault core (i.e., low‐ and high‐strain domains) of the BFZ overprints the well‐developed sub‐vertical magmatic foliation of the Jurassic meta‐diorites belonging to the Bolfin Complex. The axes are in scale *X*:*Y* = 1:2 (i.e., vertical exaggeration of 2×). Google Earth imagery is used as base reference map. Coordinate reference system and projection are WGS84 UTM Zone 19S and Transverse Mercator, respectively. Unmapped areas represent Miocene to Quaternary continental deposits.

**Figure 7:**
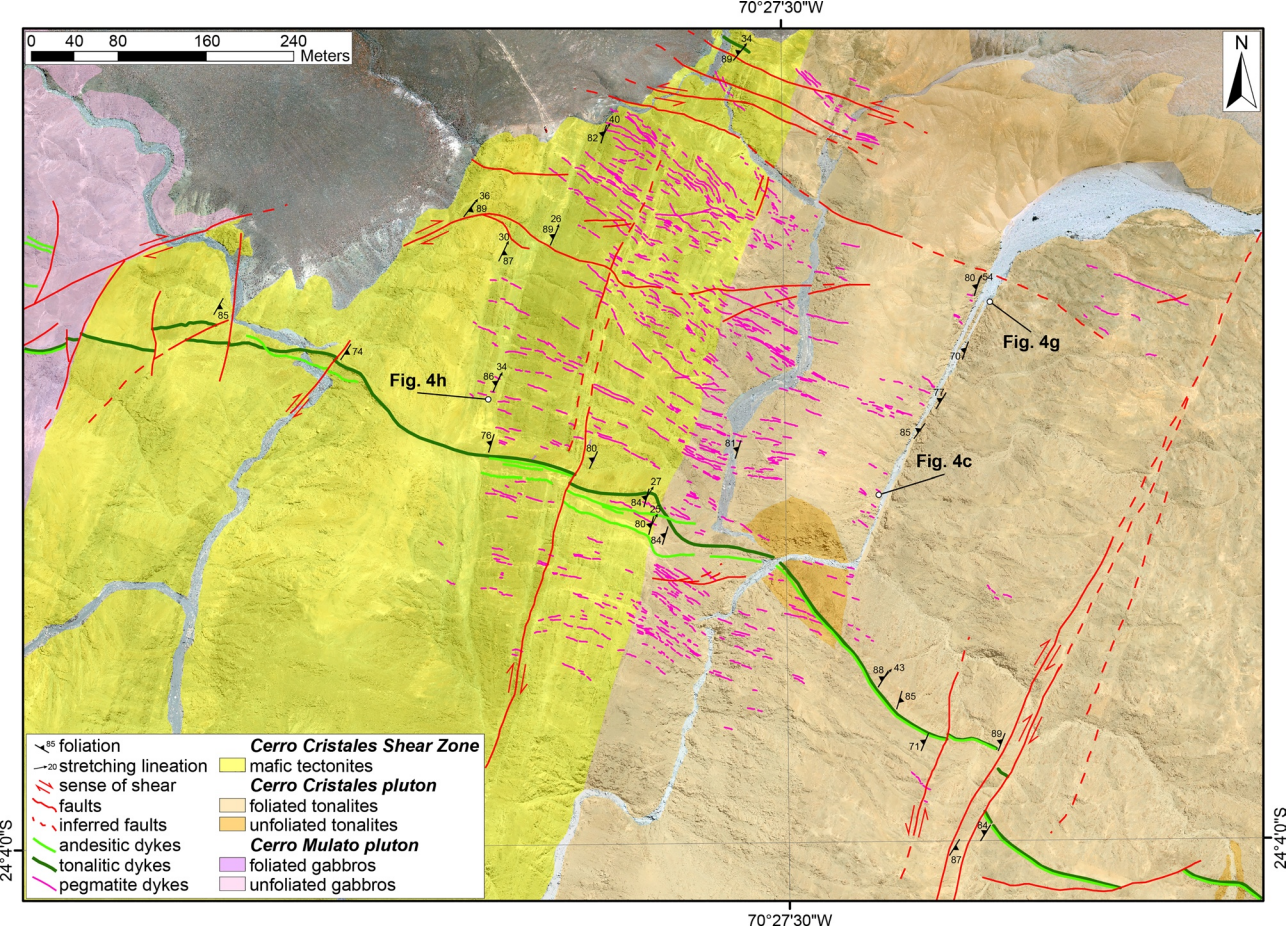
Detailed geologic map of CCSZ‐North locality along the Cerro Cristales Shear Zone. Google Earth imagery as base reference map. Coordinate reference system and projection are WGS84 UTM Zone 19S and Transverse Mercator, respectively. Unmapped areas represent Miocene to Quaternary continental deposits.

**Figure 8:**
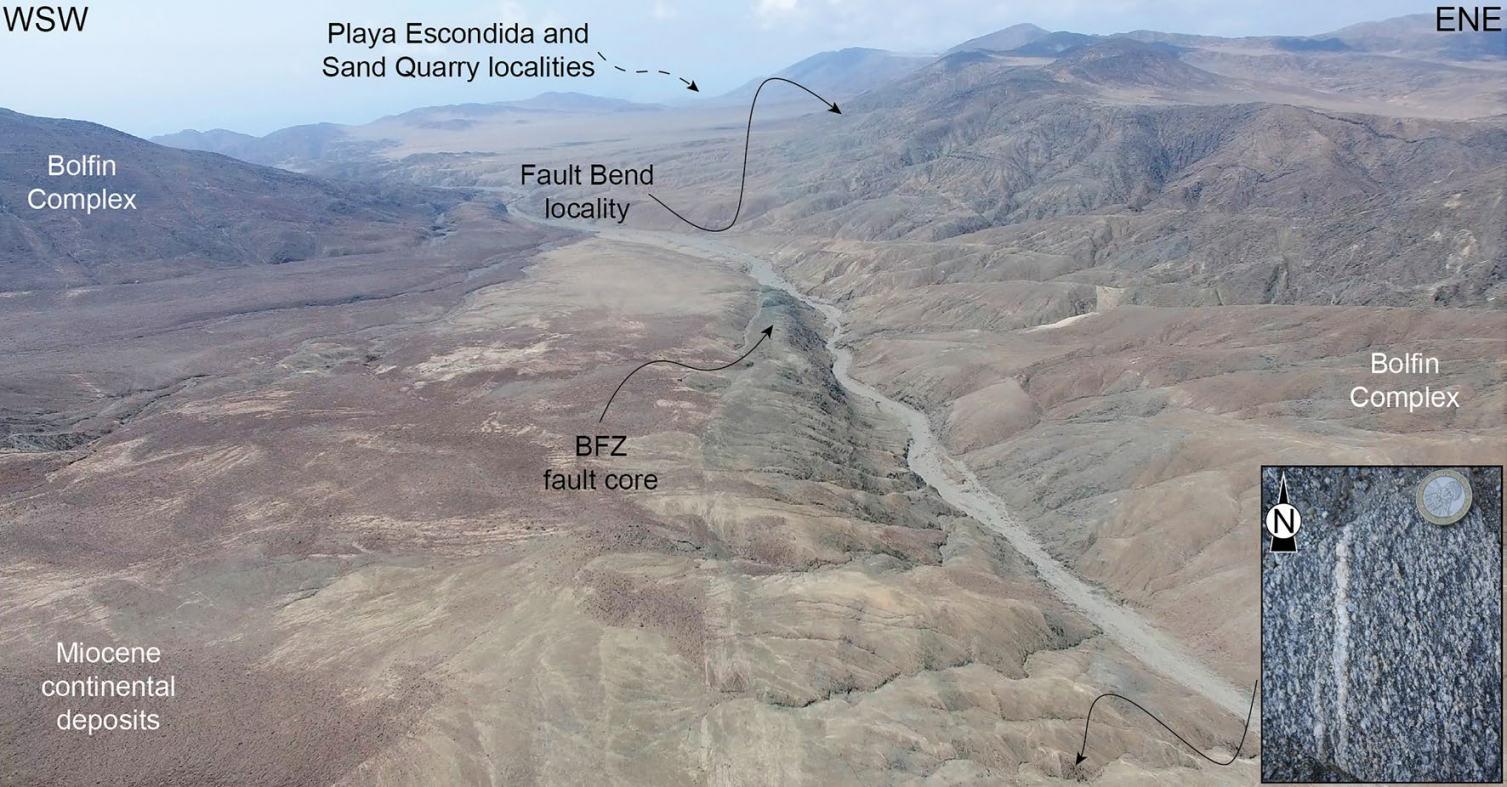
The exceptional exposure of the Bolfin Fault Zone in the Atacama Desert. The BFZ fault core overprints the well‐developed magmatic foliation of the meta‐diorites of the Bolfin Complex along the central fault segment (see detail in the bottom right, for location see the black arrow) (drone photo taken to the north of Quebrada Corta locality, see Figure 6).

Four generations of dykes were recognized based on their composition and crosscutting relationships (Figure 4). From the oldest to the youngest, they include:

1. *Amphibolitic dykes* are composed of amphibole and minor plagioclase with grain size of up to 5 mm. The dykes, up to 50‐cm‐thick, have wavy boundaries or magma‐mingling structures with the host rocks (Figure 4a) and are commonly foliated. They strike preferentially NE‐SW to NW‐SE with moderate to sub‐vertical dip angles (>45°) (Figure 5a) and intrude both the plutonic rocks of the Bolfin Complex and of the Cerro Cristales Pluton, and the fault rocks of the CCSZ.
2. *Leucocratic dykes* are composed of plagioclase with minor amphibole with grain size up to 15 mm. The dykes, up to 50‐cm‐thick, exhibit sharp boundaries with the host rocks and commonly localized ductile (solid‐state) shearing along their boundaries (Figure 4b). The dykes are steeply dipping (>80°) and strike preferentially E‐W; instead, a minor set strikes N‐S (Figure 5b).
3. *Pegmatite and aplite dykes* are widespread along both the CCSZ and the BFZ. These dykes are zoned (with K‐feldspar margins and quartz‐plagioclase‐muscovite cores), have sharp boundaries with the host rocks, and do not show an internal ductile fabric (Figures 4c and 4d). Pegmatites, up to ∼50‐cm‐thick (Figure 4c), are arranged into two sets striking to NE and NW (the latter set is more pervasive) (Figure 5d). Steeply dipping (>70°) pegmatites cut the CCSZ (Figures 4c and 7). Aplites, up to 5‐cm‐thick (Figure 4d), are arranged into three main sets, moderately to shallowly dipping toward NE, SW, and SE (Figures 4d and 5d). Pegmatites and aplites cut each other and cut the amphibolitic and leucocratic dykes (Figures 4a, and 4c–4d).
4. *Andesitic and tonalitic dykes* have sharp contacts with the host rocks and do not show ductile internal deformation. The dyke contacts are locally exploited by brittle faults. The andesitic dykes, the most common dykes in the Bolfin area, have lengths up to several kilometers and widths up to ∼3 m (Figures 6, 7, 8). The few tonalitic dykes have lengths up to hundreds of meters and widths up to 4 m (Figures 6 and 7). The andesitic dykes cut the CCSZ and are cut by the BFZ (Figures 6 and 7). There are several sets of andesitic and tonalitic dykes (Figure 5e). The steeply (>70°) and gently (<60°) dipping NW‐striking andesitic dykes cut: (a) the CCSZ, (b) the pegmatite and aplite dykes, (c) the NE‐striking andesitic to tonalitic dykes, and (d) the gently dipping (<60°) NW‐striking tonalitic dykes (at CCSZ‐North, Figure 7). Along the BFZ (in the Ni Miedo area, Figure 3), the NW‐striking tonalitic dykes cut the NE‐striking andesitic to tonalitic dykes. In conclusion, the andesitic and tonalitic dyke cut each other and cut all the other generations of dykes.

### 4.2 Magmatic and Solid‐State Deformation

#### 4.2.1 Cerro Cristales Shear Zone

The CCSZ is a 30‐km‐long and up to ∼600‐m‐thick shear zone bounding the eastern and southern sides of the ellipse‐shaped Cerro Cristales Pluton (Figure 2). The CCSZ mainly strikes NNE‐SSW, bending toward E‐W in its southern end (Figure 2). At Quebrada Museo locality (Figures 2 and 6), the meta‐diorite shows a steep (>75°) NNE‐striking magmatic foliation defined by alignment of feldspar and amphibole crystals. The contact between the CCSZ and the Bolfin Complex is transitional and highlighted by a swarm of elongate mafic microgranular enclaves sub‐parallel to the magmatic foliation (Figure 4e). The CCSZ consists of strongly foliated and lineated high‐grade rocks (hereafter referred to as mafic tectonites). The foliation of the mafic tectonites is steeply dipping (>80°) and parallel to the magmatic foliation with a prominent stretching lineation, marked by amphibole, plunging shallowly (<30°) to NNE (Figure 2). Kinematic indicators indicate dextral sense of shear (Figure 4f).

At CCSZ‐North locality (Figures 2 and 7), the Cerro Cristales tonalite is strongly foliated (Figure 4g). The sub‐vertical magmatic foliation, marked by alignment of euhedral plagioclase and amphibole crystals, strikes sub‐vertically (>80°) NNE‐SSW and is associated with a mineral lineation, marked by quartz aggregates, plunging shallowly toward NNE (Figure 2). Close to the contact with the CCSZ, the presence of asymmetric mafic microgranular enclaves within the Cerro Cristales tonalite indicate a dextral sense of shear (Figure 4g). The contact between the Cerro Cristales tonalite and the mafic tectonites of the CCSZ is sharp (Figure 7). The mafic tectonites show the same structural features as observed at Quebrada Museo locality (Figures 2 and 4h).

#### 4.2.2 Magmatic Foliation Along the Bolfin Fault Zone

The rocks of the Bolfin Complex and Cerro Cristales Pluton are foliated along most of the BFZ. The magmatic foliation is defined by the preferred alignment of plagioclase and amphibole crystals (Figure 8), and by elongated mafic enclaves. From north to south (localities, geologic maps, and structural data in Figure 3), the magmatic foliation:

- strikes N‐S and dips steeply to the E (Bolfin Complex, Playa Escondida and Sand Quarry localities);
- strikes N‐S and is sub‐vertical (Bolfin Complex, Fault Bend locality);
- strikes N‐S, is sub‐vertical and ∼4‐km‐long and ∼300‐m‐wide (Bolfin Complex, Quebrada Corta area; Figures 6 and 8);
- strikes NW‐SE to NNW‐SSE and is sub‐vertical (Cerro Cristales Pluton, Ni Miedo locality);
- is weak and scattered (the plutonic rocks are mainly isotropic), and strikes ENE‐WSW with moderate to shallow dip angles (<40°) toward S or NNW (Cerro Cristales Pluton, Quebrada Larga locality).

#### 4.2.3 Small‐Scale Ductile Shear Zones

Small‐scale (cm‐dm thick) ductile shear zones are common in the studied area. Paired shear zones (*sensu* Pennacchioni & Mancktelow, 2018) flank sub‐vertical leucocratic dykes in the Bolfin Complex (Figure 4b). These strike‐slip shear zones accommodated either dextral (E‐striking set) or sinistral (N‐striking to NW‐striking set) displacement (Figure 5c) of as much as 1 m. Some of the N‐striking to NW‐striking amphibolitic dykes localized sinistral, strike‐slip ductile shearing (Figure 5c) with development of internal S‐C foliation and sigmoidal amphiboles.

### 4.3 Brittle Deformation

#### 4.3.1 Brittle Overprint of the Cerro Cristales Shear Zone

At CCZS‐North locality (Figures 2 and 7), brittle faults occur in two sets striking NNE and W‐to‐NW, respectively. The NNE‐striking faults overprint the foliation of both the Cerro Cristales Pluton and the mafic tectonites of the CCSZ, and crosscut the NW‐striking dykes (Figure 7). The fault rocks consist of dark green, massive cataclasites bounding light green fault gouges, up to tens of centimeters thick. The few measured chlorite‐bearing slickenlines in cataclasites are shallowly plunging to NNE. The presence of dykes offset by cataclasites indicates dextral strike‐slip kinematics.

The W‐to‐NW‐striking fault set dips gently (>45°) toward N to NE. This set consists of (i) dark green cataclasites and (ii) lineated fault surfaces. The cataclasites are commonly associated with brownish‐colored pseudotachylytes, similar to those found along the BFZ s.s (Section 4.3.2). Riedel‐type structures indicate dextral strike‐slip kinematics. In contrast, the lineated fault surfaces show well‐developed epidote‐bearing slickenfibers indicating mainly normal dip‐slip kinematics, with measured displacement of as much as 1 m. Locally, red‐colored fault gouges exploit the W‐striking to NW‐striking faults. The fault gouges consist of palygorskite, calcite, gypsum, and hematite in variable modal proportions (XRPD‐RIR analysis; Section 5.3) and are associated with the Late Cenozoic extensional reactivation (Section 4.3.3).

#### 4.3.2 Bolfin Fault Zone Sensu‐Strictu (BFZ s.s.)

**Figure 9:**
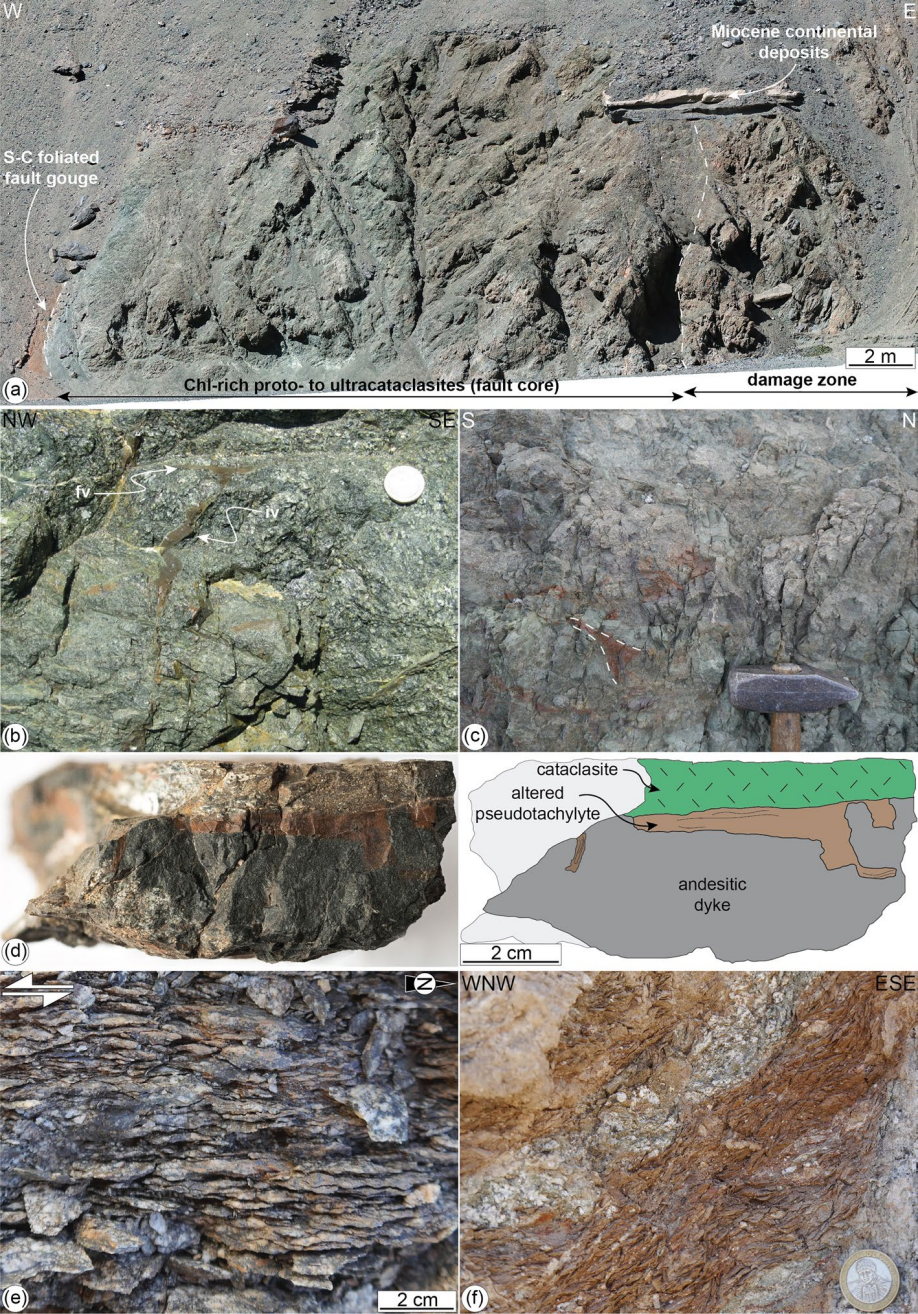
Brittle structures along the Bolfin Fault Zone (**deformation stages 4 and 5**). (a) Fault core section nearly orthogonal to fault strike. The fault core (∼17‐m‐wide) includes green chlorite‐rich protocataclasites to ultracataclasites (**deformation stage 4**). To the left, the reddish, foliated fault gouge (**deformation stage 5**) overprints the chlorite‐rich cataclasites (Sand Quarry locality) and puts in contact the Miocene continental deposits with the crystalline rocks. WGS84 GPS location: 23.8831611°S, 70.4880389°W. (b) Pseudotachylyte fault vein (fv) with cm in size injection vein (iv) intruding protobreccias and cataclasites of the fault core (**deformation stage 4**, Playa Escondida locality). 100‐pesos coin for scale. WGS84 GPS location: 23.8498611°S, 70.5032555°W. (c) Massive green cataclasites at the fault core of the BFZ. Multiple generations of brown to black, dismembered, and altered pseudotachylytes are found in the fault core (**deformation stage 4**, Ni Miedo locality). Hammer for scale; head width: 12 cm. WGS84 GPS location: 24.0796167°S, 70.4270750°W. (d) Brownish pseudotachylyte fault vein at the contact between an andesitic dyke and green cataclasite, and sketch (**deformation stage 4**, samples 19–98 from the damage zone at Quebrada Larga locality). WGS84 GPS location: 24.1732900°S, 70.3903500°W. (e) S‐C and S‐C′ brittle‐ductile shear zones spatially and kinematically (same sinistral strike‐slip shear sense) associated with the BFZ fault core (**deformation stage 4**, Fault Bend locality). WGS84 GPS location: 23.9430930°S, 70.462819°W. (f) Red foliated fault gouge associated with the late Cenozoic extension exploiting and overprinting the chlorite‐rich cataclasites within the BFZ core (**deformation stage 5**, Ni Miedo locality). 100‐pesos coin for scale. WGS84 GPS location: 24.0744450°S, 70.4306630°W.

The BFZ includes multiple fault core strands, up to 5‐m‐thick each, over a zone as wide as 75 m (Figures 6 and 8). The fault core strands (high‐strain cataclastic domains) consist of dark green to black cataclasites and ultracataclasites (Figures 6 and 9a–c), transitionally or sharply bounding low‐strain cataclastic domains of dark green protobreccias to protocataclasites, where the original magmatic fabric of the host rocks is still recognizable. Thin anastomosing bands of cataclasites are commonly observed within the low‐strain domains. The cataclastic rocks are cemented by chlorite and minor epidote (Section 5.2). The cataclasites and ultracataclasites are either massive or foliated with an S‐C fabric indicating sinistral strike‐slip kinematics (Figures 9a–9c). Exposed slickenlines are rare and plunge shallowly toward NNW to N. From north to south (localities, geologic map, and structural data in Figure 3), the sinuous fault core of the BFZ:

- strikes ∼NW‐SE and dips toward SW sub‐parallel to the magmatic foliation (Bolfin Complex, Playa Escondida locality);
- strikes N‐S and dips toward W sub‐parallel to the magmatic foliation (Bolfin Complex, Sand Quarry locality);
- bends from NNW‐SSE to N‐S, is sub‐vertical (>80°) and partially overprints the magmatic foliation (Bolfin Complex, Fault Bend locality);
- strikes N‐S, is sub‐vertical and exploits the ∼4‐km‐long magmatic foliation (Bolfin Complex, Quebrada Corta locality; Figures 6 and 8);
- strikes N‐S, is sub‐vertical and exploits the magmatic foliation (Cerro Cristales Pluton, Ni Miedo locality);
- is poorly exposed toward the fault linkage with the Caleta Coloso Fault (Cerro Cristales Pluton, Quebrada Larga locality).

Brittle deformation within the damage zone is accommodated by dark green cataclasites and sharp chlorite‐bearing lineated slip surfaces. These structures are either oriented sub‐parallel to the fault core or strike NW‐SE. The latter corresponds to Riedel‐type and splay faults of the BFZ related to the large‐scale Caleta Coloso Duplex (Cembrano et al., 2005). Steeply dipping brittle‐ductile shear zones with a composite S‐C and S‐C′ foliation, indicating sinistral strike‐slip kinematics, are discontinuously present within the BFZ fault core (Figure 9e). These foliated fault rocks are up to 1‐m‐thick and make the transition to both the green cataclasites of the fault core strands and the foliated damaged host rocks.

Pseudotachylyte veins (brownish to black in color and up to 2 cm in thickness) occur along the BFZ (Figures 9b–9d), especially in the fault core. The pseudotachylytes are polyphase and dismembered (Figure 9d). In the Quebrada Larga locality, pseudotachylytes are common in subsidiary faults across the damage zone and decorate the contact between green cataclasites and NE‐to E‐dipping andesitic dykes (Figure 9d). Here, pseudotachylyte reactivation is rare.

#### 4.3.3 Cenozoic Shallow Extensional Faulting

Fault gouges, discrete faults, and calcite‐bearing veins either exploit or cut the cataclasites both in the fault core and in the damage zone of the BFZ (Figures 3, 9a, and 9f) (see also Olivares et al., 2010). The fault gouges consist of palygorskite, calcite, halite, gypsum, and hematite in variable modal proportions (Table 1), and show S‐C composite foliations consistent with an extensional kinematics (Figure 9f). The discrete faults have calcite‐bearing and hematite‐bearing slickenlines and slickenfibers on the fault surface and occur in two sets: (i) an NW‐striking to NNE‐striking extensional to dextral‐transtensional set, and (ii) an E‐striking to ESE‐striking, extensional to sinistral‐transtensional set (Figure 3).

**Table 1:** Modal Composition of Host Rocks and Fault Zone Rocks From XRPD‐RIR Semi‐Quantitative Analysis

Locality	Rock type	Qz	Pl	Kfs	Amp	Act	Bt/Ms	Chl	Ep	Cal	Spl	Gp	Plg	Hl	Hem/Ant	Others
PE	Altered meta‐diorite	15	36	4	28	3		13								
PE	Cataclasite	27	38			16	≤1	7	3	3						5^a^
PE	Cataclasite	39	35	2		≤1		12	3	4						3^a^
PE	Cataclasite	40	36	5				5	7	3						2^a^
PE	Foliated cataclasite	19	27	7		7		15	25							
PE	Fault gouge	7				≤1		≤1	11	39		14	20	6		
SQ	Altered meta‐diorite	37	20	7		8		10	16							2^a^
SQ	Fault gouge	16	26	5		≤1		7	11	6		12	15	≤1		
SQ	Fault gouge							2	≤1	33		5	42	17		
SQ	Foliated fault gouge							5				≤1	75	19		
SQ	Fault gouge	10	49		12			4	6	19			≤1			
QC	Meta‐diorite		67	4	9		7	7	5		≤1					≤1^b^
QC	Altered meta‐diorite	8	52		19	11		9								≤1^b^
QC	Sheared amphibolitic dyke		26		55			14	2							4^c^
QC	Fault gouge	74				5		≤1	≤1	18						
QC	Fault gouge	46						2		49					3	
QC	Foliated fault gouge	36						16		8		16		3	3	18^d^
QL	Altered granodiorite	27	42	11		3	6	11								
QL	Foliated cataclasite	39	26	4			≤1	19	9	4						
QL	Foliated cataclasite	31	38	13			3	13		2						
QM	Mafic tectonites	≤1	43		44			8			3					
QM	Sheared amphibolitic dyke	≤1	35		52			9			3					

*Note*. PE: Playa Escondida; SQ: Sand Quarry; QC: Quebrada Corta; QL: Quebrada Larga; QM: Quebrada Museo. Mineral abbreviations after Whitney and Evans (2010).

^a^ Tnt.

^b^ Crd.

^c^ Cpx.

^d^ Ilt.

## 5 Microstructural Observations

We describe the microstructures of the ductile and brittle features pertaining to the CCSZ and BFZ s.s. The XRPD‐RIR and EMPA analyses of the fault zone rocks and the mineral phases are reported in Tables 1 and 2, respectively.

**Table 2:** Mineral Phase Compositions of Host Rocks and Fault Zone Rocks as Obtained From EMPA Analysis

Locality	Quebrada Museo													
Rock type	Mafic tectonites						Localized ductile shear zones							
Mineral phase	Pl		Amp		Ilm		Pl1		Pl2		Amp		Mag	
No. of point analysis	9	s.d.	9	s.d.	2	s.d.	3	s.d.	7	s.d.	13	s.d.	2	s.d.
Component (wt%)														
Na_2_O	5.27	0.31	1.14	0.24	0.08	0.03	5.93	0.13	7.42	0.17	0.84	0.47	0.03	0.00
MgO	0.09	0.04	13.09	0.49	0.23	0.01	0.06	0.03	0.16	0.21	11.71	1.87	0.20	0.01
Al_2_O_3_	29.05	0.56	9.03	0.83	0.14	0.05	27.66	0.18	25.44	0.53	5.71	2.43	0.08	0.00
SiO_2_	54.23	0.66	45.51	1.12	0.06	0.00	56.01	0.29	59.39	0.34	48.12	3.34	0.08	0.03
K_2_O	0.13	0.05	0.67	0.11	0.04	0.00	0.23	0.03	0.15	0.04	0.45	0.26	0.02	0.02
CaO	10.70	0.33	11.87	0.28	0.11	0.00	9.50	0.21	6.61	0.26	11.44	0.42	0.23	0.01
TiO_2_	0.02	0.03	1.53	0.26	50.48	0.57	0.04	0.04	0.03	0.03	0.79	0.51	0.00	0.00
Cr_2_O_3_	0.02	0.03	0.03	0.03	0.08	0.00	0.01	0.02	0.02	0.02	0.03	0.03	0.08	0.01
MnO	0.02	0.02	0.29	0.08	4.00	0.12	0.01	0.01	0.04	0.04	0.53	0.08	0.07	0.04
FeO	0.24	0.07	13.96	0.54	44.53	0.50	0.16	0.07	0.26	0.24	18.02	1.96	92.28	1.21
Total	99.77	0.21	97.11	0.40	99.74	0.13	99.63	0.10	99.52	0.19	97.64	0.42	93.08	0.13

Locality	Playa Escondida													
Rock type	Cataclasites						Pst with vesicles							
Mineral phase	Chl		Ep		Act		Ab matrix		Kfs matrix		Pl clasts		Amp microlites	
No. of point analysis	16	s.d.	3	s.d.	2	s.d.	3	s.d.	16	s.d.	3	s.d.	5	s.d.
Component (wt%)														
Na_2_O	0.02	0.03	0.00	0.01	0.18	0.03	7.56	0.38	1.52	0.96	8.32	0.54	1.85	0.39
MgO	15.03	0.53	0.02	0.01	17.37	0.07	0.09	0.10	0.41	0.29	0.08	0.02	8.62	2.73
Al_2_O_3_	19.56	0.78	21.65	0.23	2.68	1.61	24.39	0.39	17.90	1.01	23.67	0.51	13.55	1.83
SiO_2_	26.55	0.79	37.33	0.26	53.16	2.48	60.14	0.31	64.86	1.94	63.10	0.86	47.23	4.86
K_2_O	0.05	0.07	0.00	0.00	0.05	0.01	0.55	0.27	12.53	2.50	0.30	0.17	2.95	2.01
CaO	0.09	0.08	23.12	0.23	11.59	1.28	6.01	0.31	1.08	1.04	5.31	0.62	6.48	1.39
TiO_2_	0.05	0.02	0.09	0.05	0.05	0.04	0.06	0.03	0.19	0.19	0.05	0.02	1.97	1.01
Cr_2_O_3_	0.02	0.02	0.02	0.02	0.02	0.00	0.00	0.00	0.01	0.02	0.00	0.00	0.00	0.00
MnO	0.48	0.05	0.13	0.05	0.32	0.04	0.02	0.01	0.03	0.03	0.03	0.03	0.34	0.10
FeO	26.17	0.58	14.27	0.18	12.22	1.23	0.64	0.43	1.34	0.74	0.37	0.07	14.34	3.38
Total	88.02	0.30	96.64	0.10	97.65	0.68	99.45	0.40	99.85	1.01	101.23	0.83	97.33	1.51

Locality	Sand Quarry										Fault Bend			
Rock type	Vein cut by Pst		Altered Pst								Host rock		Pst	
Mineral phase	Chl		Chl		Ab microlites		Kfs matrix		Tnt		Chl		Ep	
No. of point analysis	7	s.d.	12	s.d.	49	s.d.	16	s.d.	5	s.d.	19	s.d.	6	s.d.
Component (wt%)														
Na_2_O	0.05	0.03	0.05	0.04	7.82	2.01	0.77	0.77	3.81	4.07	0.16	0.23	0.03	0.02
MgO	17.20	0.42	17.48	0.33	0.65	1.20	0.11	0.04	0.26	0.31	17.45	2.49	0.11	0.03
Al_2_O_3_	19.85	0.63	20.66	0.33	17.42	2.95	17.63	0.94	15.20	3.70	17.97	1.59	22.84	0.52
SiO_2_	27.18	0.82	26.65	0.50	65.35	6.32	66.14	1.99	56.24	6.71	29.94	2.20	37.42	0.12
K_2_O	0.02	0.01	0.03	0.02	2.24	1.92	14.97	1.60	6.86	5.04	0.50	0.66	0.02	0.01
CaO	0.15	0.08	0.10	0.04	2.03	1.61	0.18	0.31	7.11	5.70	0.73	1.15	22.92	0.18
TiO_2_	0.05	0.03	0.06	0.04	0.62	1.62	0.02	0.03	8.09	7.52	0.88	1.02	0.09	0.07
Cr_2_O_3_	0.03	0.03	0.02	0.02	0.00	0.00	0.00	0.00	0.00	0.00	0.03	0.02	0.04	0.05
MnO	0.57	0.07	0.58	0.04	0.04	0.05	0.02	0.03	0.04	0.02	0.46	0.09	0.24	0.06
FeO	23.58	1.08	23.05	0.38	3.50	2.19	0.25	0.19	2.00	2.30	21.13	2.82	13.65	0.55
Total	88.68	0.76	88.68	0.58	99.68	1.99	100.10	1.39	99.62	3.54	89.24	1.17	97.34	0.25

Locality	Fault Bend		Quebrada Corta						Quebrada Larga					
Rock type	Pst		Host rock		Cataclasites		Vein cut by Pst		Host rock					
Mineral phase	Chl		Pl		Chl		Chl		Amp		Bt		Chl	
No. of point analysis	12	s.d.	2	s.d.	3	s.d.	8	s.d.	3	s.d.	20	s.d.	28	s.d.
Component (wt%)														
Na_2_O	0.04	0.02	8.65	0.14	0.04	0.03	0.09	0.12	0.47	0.03	0.09	0.05	0.07	0.05
MgO	17.75	1.20	0.13	0.06	18.46	0.15	19.00	1.25	13.08	0.41	11.09	2.21	15.38	2.27
Al_2_O_3_	20.99	0.68	23.64	0.42	18.59	0.08	17.43	0.92	4.24	0.47	15.63	3.21	17.53	3.10
SiO_2_	26.39	0.88	62.89	1.06	28.98	0.29	30.00	1.33	50.13	0.45	35.95	2.25	30.63	3.45
K_2_O	0.05	0.04	0.20	0.07	0.06	0.01	0.05	0.03	0.29	0.08	8.18	1.86	2.23	2.43
CaO	0.15	0.05	4.02	0.40	0.38	0.18	0.58	0.48	11.64	0.32	0.24	0.43	0.48	1.06
TiO_2_	0.07	0.04	0.01	0.01	0.40	0.24	0.13	0.09	0.27	0.13	3.04	0.90	1.39	1.63
Cr_2_O_3_	0.02	0.02	0.06	0.03	0.07	0.02	0.03	0.03	0.02	0.01	0.04	0.04	0.03	0.03
MnO	0.46	0.07	0.00	0.00	0.47	0.01	0.50	0.09	0.64	0.01	0.42	0.09	0.50	0.13
FeO	22.42	1.61	0.30	0.07	20.92	0.52	20.67	1.24	15.79	0.31	20.02	3.58	21.17	1.84
Total	88.34	0.86	99.89	0.22	88.37	0.08	88.48	0.63	96.58	0.35	94.70	1.91	89.42	1.56

Locality	Quebrada Larga							
Rock type	Cataclasites				Altered Pst			
Mineral phase	Act		Chl		Kfs matrix		Chl	
No. of point analysis	3	s.d.	13	s.d.	13	s.d.	8	s.d.
Component (wt%)								
Na_2_O	0.31	0.13	0.07	0.05	0.61	0.39	0.04	0.03
MgO	13.15	0.97	16.15	1.87	0.11	0.07	15.65	0.85
Al_2_O_3_	3.16	0.64	17.55	2.63	19.06	0.26	22.31	0.80
SiO_2_	51.61	1.49	30.05	2.82	63.99	0.31	26.76	1.15
K_2_O	0.18	0.07	1.29	1.42	16.22	0.71	0.43	0.65
CaO	12.02	0.49	0.72	1.45	0.06	0.10	0.13	0.03
TiO_2_	0.09	0.05	1.08	1.63	0.06	0.03	0.14	0.06
Cr_2_O_3_	0.03	0.04	0.03	0.03	0.04	0.04	0.04	0.03
MnO	0.72	0.11	0.47	0.11	0.02	0.03	0.63	0.07
FeO	16.00	0.86	20.88	2.03	0.32	0.13	22.54	1.05
Total	97.27	1.47	88.29	0.63	100.47	0.21	88.68	1.02

*Note*. Pst, pseudotachylytes. Mineral abbreviation after Whitney & Evans (2010).

### 5.1 Magmatic and Solid‐State Deformation (*T* > 700°C)

**Figure 10:**
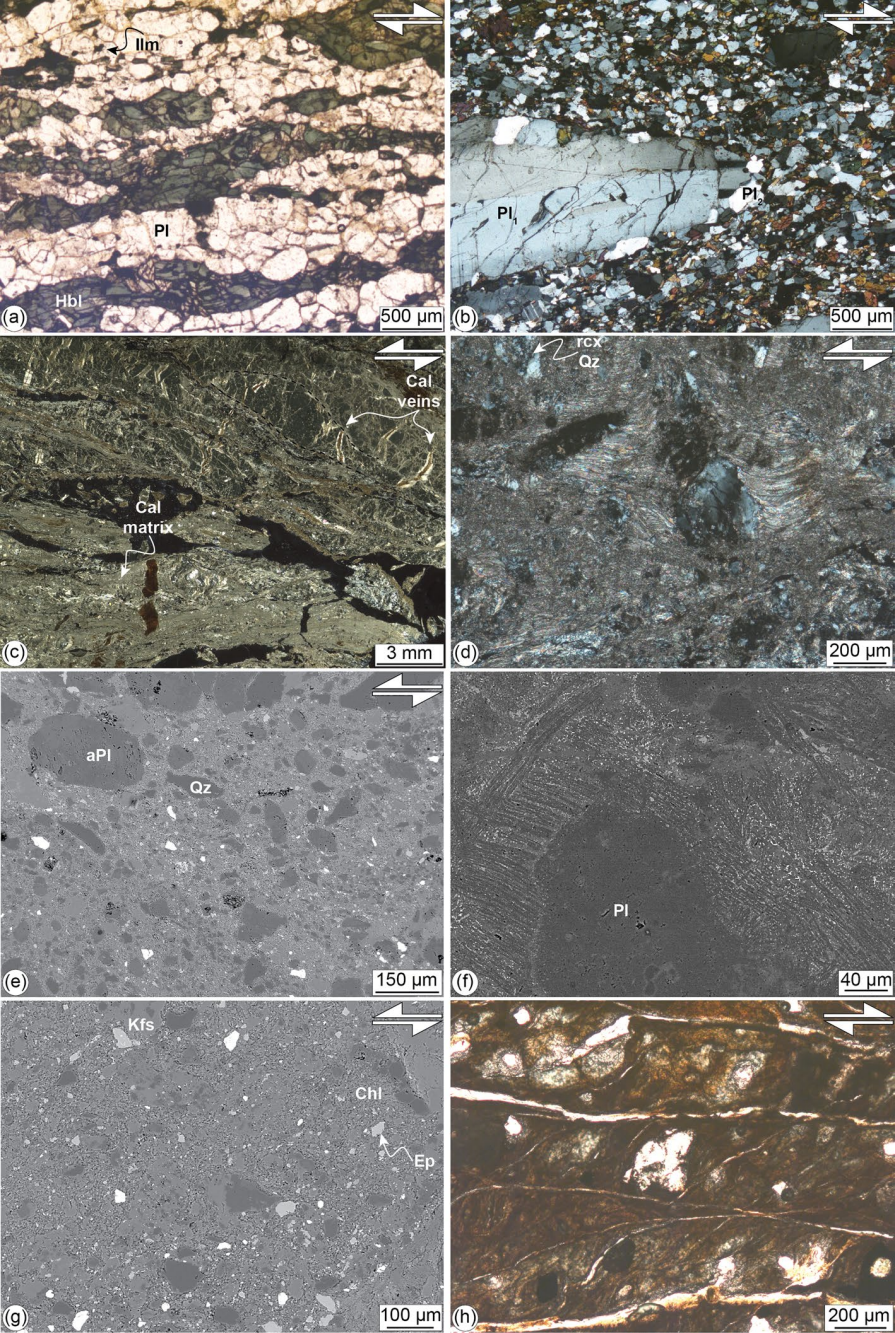
Microstructures of the fault zone rocks from the Bolfin area. Mineral abbreviations: Pl = plagioclase, aPl = altered plagioclase, Hbl = hornblende, Cal = calcite, Qz = quartz, rcx Qz = recrystallized quartz, Chl = chlorite, Ep = epidote, Ilm = ilmenite. (a) Polygonal aggregates of recrystallized hornblende and plagioclase forming the mafic tectonites of the CCSZ (**deformation stage 1**). Sigmoidal amphibole indicates dextral (top‐to‐SSW) sense of shear. Parallel‐polarized light microphotograph, samples 19–25. WGS84 GPS location: 23.9966700°S, 70.4394900°W. (b) Localized ductile shear zone (**deformation stage 2**). Centimeter in size plagioclase porphyroclast (Pl_1_) with undulose extinction shows mantle recrystallized into finer grained grains (Pl_2_). The porphyroclast is hosted in fine‐grained matrix including equigranular plagioclase and amphibole. Opaque mineral is magnetite present at triple grain junctions. Cross‐polarized light microphotograph, sample B09‐19. WGS84 GPS location: 23.99998°S, 70.444594°W. (c). Brittle‐ductile shear zone with sinistral sense of shear (**deformation stage 4**). Microlithons consist of millimeter in size altered plagioclase (medium gray) delineated by micro‐stylolite seams (dashed black lines). The latter defines an S‐C foliation indicating sinistral kinematics consistently with the orientation of the crack‐seal calcite veins (Cal veins). High‐strain horizons (light gray) consist of quartz porphyroclasts hosted in fine‐grained matrix of calcite. Cross‐polarized light thin section scan, sample FB20‐676. WGS84 GPS location: 23.942990°S, 70.462819°W. (d). Strain fringes of fibrous calcite around a quartz porphyroclast. The fringe structure indicates sinistral strike‐slip kinematics. The quartz porphyroclasts show undulose extension and are recrystallized into fine‐grained aggregates along grain boundaries and micro‐fractures. Cross‐polarized light microphotograph, sample FB20‐676. (e) Cataclasite from the BFZ (**deformation stage 4**). Clasts consist mainly of quartz and altered plagioclase. The light‐gray matrix includes chlorite, epidote, and K‐feldspar. BSE‐SEM image, samples 19–86. WGS84 GPS location: 24.1753100°S, 70.3901400°W. (f) Microlites of plagioclase (medium gray) and K‐feldspar (dark gray), and interstitial acicular biotite and titanite (bright color) in a poorly altered pseudotachylyte fault vein (**deformation stage 4**). The microlites wrap a plagioclase clast with spinifex microstructure. BSE‐SEM image, sample SQ09‐18. WGS84 GPS location: 23.8830972°S, 70.4880111°W. (g). Altered pseudotachylyte (**deformation stage 4**). The pseudotachylyte fault vein is altered into a fine‐grained chlorite, epidote, albite, and K‐feldspar. BSE image, samples 19–91A. WGS84 GPS location: 24.1747900°S, 70.3899600°W. (h) Fault gouge from the late Cenozoic faults (**deformation stage 5**). The gouge shows an S‐C foliation, defined by palygorskite + clay + hematite, wrapping around halite clasts. Parallel‐polarized light microphotograph, sample SQ13‐18. WGS84 GPS location: 23.8830194°S, 70.4881222°W.

*Mafic tectonites* forming the CCSZ consist of equigranular, polygonal aggregates of plagioclase and amphibole of ∼150–250 μm grain size (Figure 10a). Plagioclase ranges in composition between Ab_50_An_50_Or_0_ and, more commonly, Ab_43_An_56_Or_1_ (Table 2). Ilmenite is commonly present in the plagioclase aggregates at triple grain junctions and along grain boundaries (Figure 10a). Plagioclase is locally replaced by oligoclase + calcite + sericite. Amphibole, mostly hornblende and minor edenite (Table 2), is locally replaced by chlorite. Plagioclase‐amphibole geothermometry (Holland & Blundy, 1994; Molina et al., 2015) yields *T* = 788 ± 50°C and *P* = 185 ± 150 MPa for recrystallization.

*Localized ductile shear zones* bounding leucocratic dykes (Figure 4b) have a homogeneous recrystallized polygonal matrix (∼50 μm grain size) of plagioclase (Ab_67_An_32_Or_1_), amphibole and magnetite wrapping around mm‐sized amphibole and plagioclase (Ab_52_An_47_Or_1_) porphyroclasts (Figure 10b and Table 2).

### 5.2 Brittle Seismogenic Deformation (*T* ≤ 300°C)

*Damaged host rocks* contain pervasive microfractures and veins whose spatial density increases toward the fault core (see Gomila et al., 2016; Jensen et al., 2011; Mitchell & Faulkner, 2009 for description of nearby faults). Magmatic minerals present intense fluid‐induced alteration. Plagioclase is either altered to fine‐grained sericite + calcite ± epidote or replaced by albite (Table 2). Amphibole is replaced by either (Fe‐)actinolite or chlorite and opaques. Biotite is replaced by chlorite and opaques. Quartz shows undulose extinction and K‐feldspar is fractured and micro‐faulted. Micro‐stylolite seams are common in the damaged host rocks and are sub‐parallel to the cataclasites.

*Brittle‐ductile shear zones* consist of (i) microlithons of plagioclase (altered to fine‐grained white mica + calcite or replaced by albite), (ii) high‐strain horizons of quartz porphyroclasts immersed in a fine‐grained (<20 μm grain size) recrystallized calcite matrix, and (iii) calcite antitaxial extensional veins (Figures 10c and 10d). Altered plagioclase microlithons are defined by micro‐stylolite seams delineating a composite S‐C and S‐C′ foliation (Figure 10c). Quartz porphyroclasts (i) show undulose extinction, (ii) are locally recrystallized into fine‐grained aggregates along grain boundaries and microfractures, and (iii) are surrounded by pressure shadows of fibrous calcite (i.e., strain fringe) (Figure 10d). Antitaxial veins consist of fibrous calcite, which cut the plagioclase microlithons and the high‐strain horizons. The veins are orthogonal to the micro‐stylolite seams and their spatial arrangement is consistent with sinistral strike‐slip kinematics (Figure 10c).

*Cataclasites* consist of a fine‐grained matrix of chlorite + epidote + quartz + albite + K‐feldspar, including angular clasts of altered plagioclase, quartz, and earlier cataclasites (Figure 10e). Cataclasites are locally foliated and, in the thickest horizons, layered for variable matrix/clasts ratios.

*Pseudotachylytes* show typical features of quenched melts: chilled margins, flow structures, and presence of microlites and spherulites (e.g., Di Toro et al., 2009; Swanson, 1992). Alteration variably affected the pseudotachylytes. The most pristine pseudotachylytes have a homogeneous cryptocrystalline matrix with a “K‐feldspar‐rich” composition which contains (i) albite microlites, intergrown with amphibole and titanite (Figure 10f), locally arranged into spherulitic aggregates and (ii) quartz and plagioclase clasts (Figure 10f). Altered pseudotachylytes consist of fine‐grained (∼20–30 μm grain size) albite + chlorite + epidote ± K‐feldspar association (Figure 10g).

### 5.3 Brittle Shallow Extensional Deformation (*T* < 150°C)

In the reddish foliated fault gouges (Figure 9f), the S‐C fabric is marked by fine‐grained palygorskite, clays and hematite, which wraps halite and gypsum mantled clasts (Figure 10h). Halite mantled clasts are up to 1 mm in size and commonly fractured along cleavage planes. Gypsum clasts are up to ∼200 μm in size and show undulose extension (Figure 10h). Veins consist of either (i) blocky‐shaped calcite grains or (ii) angular clasts of calcite sealed by microcrystalline calcite and minor opaque minerals. Calcite grains and clasts show thin (<1 μm) and, locally, tabular thick (i.e., up to 10 μm) twin lamellae, classified as type I and type II twins, respectively, following the scheme of Ferrill et al. (2004).

## 6 Discussion

First (Section 6.1), we discuss the field and microstructural observations (Sections 4 and 5) that allow us to constrain the P‐T deformation conditions and to recognize a sequence of five main deformation stages. This information is required to interpret the formation of the seismogenic BFZ *sensu strictu* (125–118 Ma). Then (Section 6.2), we discuss the role of precursory structures on the evolution of the BFZ s.s. and we propose a more general model of fault growth within a heterogeneous magmatic arc.

### 6.1 P‐T Deformation Conditions and Structural Evolution of the Bolfin Fault Zone

**Figure 11:**
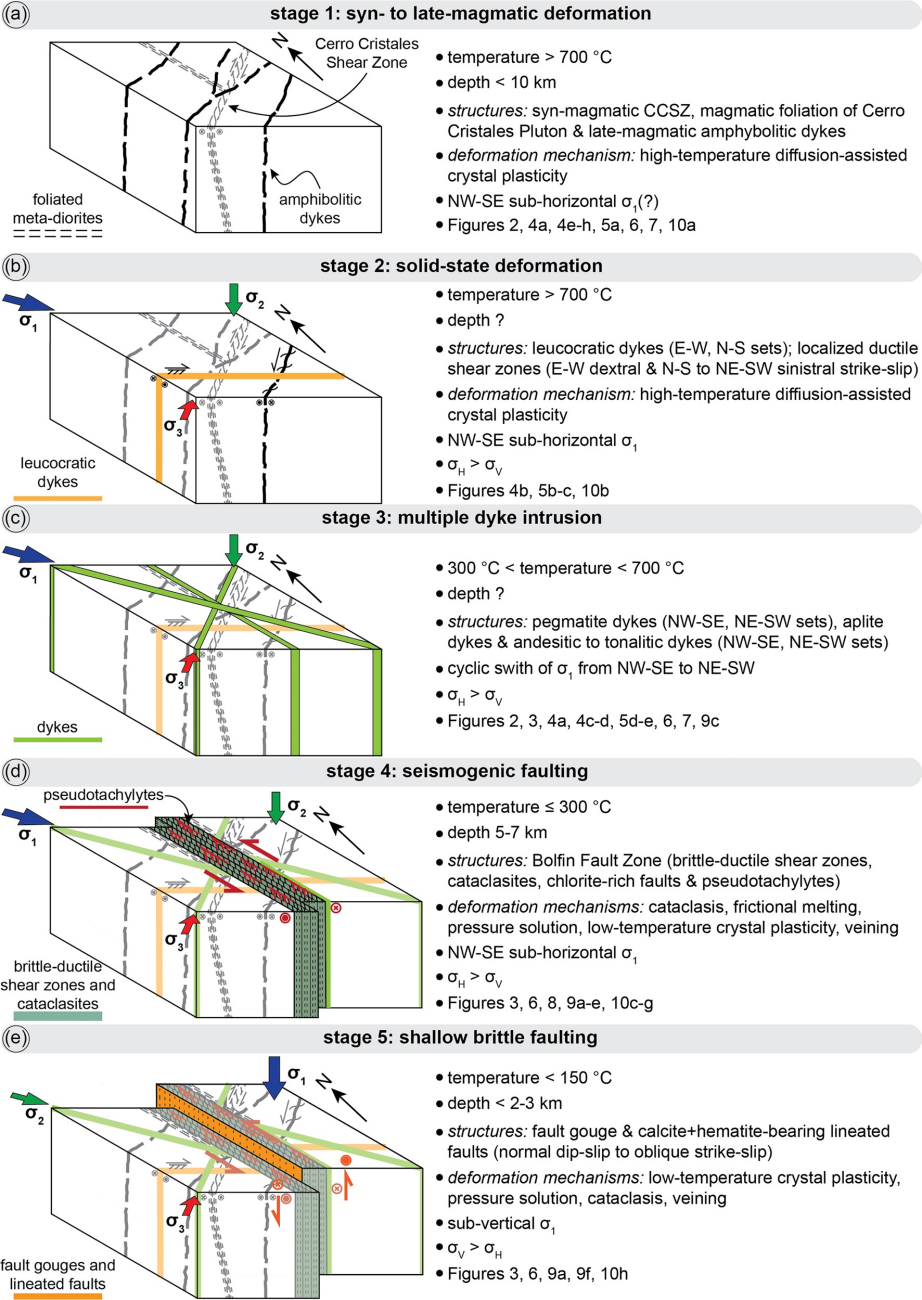
Block diagrams showing the structural evolution of the Bolfin Fault Zone (left column) and features related to each deformation stage (right column). The stress field orientation was inferred from (i) the orientation of dykes and (ii) the kinematics of ductile shear zones and faults. (a) **Deformation stage 1**: emplacement of the Cerro Cristales Pluton at shallow crustal levels (<10 km depth); formation of the large‐scale syn‐magmatic to post‐magmatic Cerro Cristales Shear Zone; emplacement of amphibolitic dykes during late‐magmatic stage. (b) **Deformation stage 2**: emplacement of leucocratic dykes and development of high‐temperature (>700°C) localized solid‐state ductile shear zones at early stage of pluton cooling. The ductile shear zones are arranged into a conjugate set (E‐W dextral strike‐slip, and N‐S to NW‐SE sinistral strike‐slip). (c) **Deformation stage 3**: emplacement of steeply dipping NE‐striking and NW‐striking pegmatite and andesite/tonalite dykes. The emplacement of these dykes suggests a mutual switch of the orientation of the maximum (*σ*_1_) and minimum (*σ*_3_) shortening directions. See main text for discussion. (d) **Deformation stage 4**: Bolfin Fault Zone s.s. The ancient (125–118 Ma) seismic activity of the BFZ is documented by widespread pseudotachylytes. Seismic faulting occurred at ≤ 300°C and 5–7 km depth in a fluid‐rich environment. (e) **Deformation stage 5**: Since the Miocene, the BFZ underwent extensional faulting at shallow crustal levels (<2–3 km depth).

The BFZ experienced a polyphase evolution that includes magmatic and solid‐state deformation episodes (stages 1–2), followed by the emplacement of multiple generations of dykes (stage 3). This predated the Early Cretaceous brittle seismogenic strike‐slip faulting (stage 4) and the late Cenozoic extensional faulting (stage 5). The whole evolution is summarized in the block diagrams of Figure 11.

#### 6.1.1 Stages 1–3: Pre‐Bolfin Fault Zone s.s. (*T* > 300°C)

##### 6.1.1.1 Stages 1–2: Syn‐Magmatic to Solid‐State Deformation (*T* > 700°C)

*Stage 1 (Syn‐magmatic to late‐magmatic deformation)*. Along the CCSZ, the orientation of the magmatic and solid‐state foliations and of the stretching lineations are similar (Figure 2). These structural features are characteristic of syn‐magmatic thermal aureoles related to pluton emplacement (Clemens, 1998; Miller & Paterson, 1999; Paterson & Vernon, 1995 and references therein). At CCSZ‐North locality, dextral strike‐slip shearing in the mafic tectonites is spatially associated with melt segregation structures (Figure 4h) (e.g., Sawyer, 2000; Weinberg, 2006). This indicates that shearing accommodated by the CCSZ initiated during crystallization of the Cerro Cristales Pluton (*T* > 700°C) as also supported by the high‐temperature conditions (788 ± 50°C) estimated for the recrystallized matrix of the mafic tectonites.

Based on our observations, we interpret the CCSZ as a large‐scale syn‐magmatic to post‐magmatic shear zone related to the emplacement of the Cerro Cristales Pluton and the pervasive magmatic foliation of the outer shell of the pluton related to magma inflation/ballooning. Additionally, the dextral slip of the CCSZ may result from the local stress perturbation induced by the pluton emplacement, which possibly perturbed, at least locally, the regional far‐stress field associated with the ancient SE‐directed oblique subduction (Scheuber & González, 1999). González (1999) estimated an emplacement depth of ∼13 km (400 MPa) for the Cerro Cristales Pluton, based on hornblende geobarometry and assuming a geothermal gradient of 30 °C/km. Instead, assuming a geothermal gradient of ∼50°C/km, typical of an active magmatic arc (e.g., the Southern Andes Volcanic Zone: Pearce et al., 2020; Sielfeld et al., 2019) and considering the estimated pressure (185 ± 150 MPa) for recrystallized matrix of the mafic tectonites, the emplacement depth of the pluton results at <10 km depth, as for plutons of similar age (140–155 Ma) emplaced along the El Salado segment of the AFS (Espinoza et al., 2014; Grocott & Taylor, 2002; Seymour et al., 2020). Moreover, the emplacement of several plutons in a short time span thermally weakens the crust facilitating pluton emplacement at shallow depths (Cao et al., 2016). We conclude that the CCSZ started forming at >700°C and < 10 km depth during the emplacement of the Cerro Cristales Pluton (Figures 11a). The Bolfin Complex and the Cerro Cristales Pluton are intruded by the amphibolitic dykes, which cut also the CCSZ. These dykes show mingling structures and evidence of remelting, indicating that they intruded in a still partly molten material during a late‐magmatic deformation stage (Figures 4a and 11a).

*Stage 2 (Solid‐state deformation)*. Solid‐state deformation is recorded by the meso‐scale paired ductile shear zones (*sensu* Pennacchioni & Mancktelow, 2018) flanking leucocratic dykes (Figure 4b). Discrete ductile shear zones nucleated on compositional and structural precursors are widely reported in several granitoid plutons and meta‐granitoids in metamorphic units elsewhere (e.g., Christiansen & Pollard, 1997; Pennacchioni, 2005; Pennacchioni & Zucchi, 2013; Segall & Simpson, 1986). These localized shear zones have similar microstructural features of the mafic tectonites forming the CCSZ (Figure 10b) and are inferred to have developed during the earliest cooling stages of the plutons.

The dextral, E‐striking ductile shear zones and the sinistral, N‐to‐NE‐striking sheared amphibolitic dykes are arranged to form a conjugate set (Figure 5c) associated with a sub‐horizontal NW‐SE compressional direction (i.e., σ_1_) (Figure 11b). This σ_1_ is consistent with the SE‐directed oblique subduction recorded in the Coastal Cordillera (Scheuber & González, 1999; Veloso et al., 2015).

Scheuber and González (1999) reported localized ductile shear zones formed at greenschist‐facies metamorphic conditions, which is not consistent with the high‐temperature conditions determined for the localized shear zones described here. The absence (or very scarce occurrence) of greenschist‐facies localized ductile shear zones can be explained by the fast eastward migration of the magmatic arc, rapid regional‐scale exhumation, and the shallow emplacement depth of plutons (<10 km depth). This likely promoted a sharp transition from high‐temperature, ductile deformation to low‐temperature, brittle faulting.

##### 6.1.1.2 Stage 3: Multiple Generation of Dykes (300°C < *T* < 700°C)

Several generations of dykes intruded the Coastal Cordillera after most of the plutons crystallization (≤147 Ma, U‐Pb zircon age from the Cerro Cristales granodiorite; Domagala et al., 2016). Based on orientation and crosscutting relationships of pegmatite and andesitic dykes (Figures 4 and 5), *σ*_1_ and *σ*_3_ directions should have cyclically switched their orientation, from NW‐SE to NE‐SW (stage 3 in Figures 11c). The σ_1_‐σ_3_ cyclic switching might be related to either (i) several intra‐arc sinistral (i.e., NW‐SE directed *σ*_1_) and dextral (i.e., NE‐SW directed *σ*_1_) deformation stages, as much as the different dyke sets crosscut each other, as proposed by Scheuber and González (1999) or (ii) intermittent transient stress rotations (i.e., switch of principal stress axes) in the upper plate induced by megathrust earthquakes (Acocella et al., 2018; Becker et al., 2018; Hardebeck & Okada, 2018; Lara et al., 2004; Lupi & Miller, 2014; Lupi et al., 2020; Mancktelow & Pennacchioni, 2020; Pérez‐Flores et al., 2016). The latter interpretation is also supported by the NNE‐striking strike‐slip faults exploiting the foliation of the CCSZ and the Cerro Cristales tonalite (Figure 7). Indeed, these foliations are well‐oriented for reactivation as dextral strike‐slip faults during the transient tectonic regime with NE‐directed σ_1_. However, the hypothesis of megathrust earthquakes‐related transient stress rotations requires further work to be tested. Finally, the moderately to shallowly dipping aplite dykes are interpreted as related to exhumation occurring during Late Jurassic and Early Cretaceous.

#### 6.1.2 Stage 4: Bolfin Fault Zone s.s. (*T* ≤ 300°C)

The BFZ fault core is spatially associated and kinematically (sinistral sense of shear) consistent with the brittle‐ductile shear zones (Figures 8, 9, and 10c and 10d). The latter structures accommodated deformation by combined pressure‐solution mechanism, incipient low‐temperature crystal plasticity and fragmentation of quartz (Figures 10c and 10d), suggesting a deformation temperature ≤ 300°C (Stipp et al., 2002), consistently with their mineral assemblage. The mutual crosscutting relationship between the calcite crack‐seal extensional veins and the composite S‐C and S‐C′ fabric indicates cyclic, transient syn‐kinematic extensional fracturing, triggered by cyclic increases of pore fluid pressure, during viscous deformation. This combined diffusive to crystal‐plastic and cataclastic deformation is typical of the viscous‐plastic to elasto‐frictional transition in presence of fluids (e.g., Snoke et al., 1998). The spatial association of the brittle‐ductile shear zones with the fault core is interpreted as the result of the transition from viscous‐plastic to elasto‐frictional rheology of the BFZ, as for other fault segments of the AFS (e.g., Grocott & Taylor, 2002; Scheuber & González, 1999; Scheuber et al., 1995; Seymour et al., 2020). The transition may have resulted from (a) different *P*‐*T* deformation conditions, also during passive exhumation, (b) variations of strain rate experienced by the BFZ, or (c) a combination of (a) and (b). However, the brittle‐ductile shear zones are found discontinuously along the BFZ. This could either reflect (a) a change of *P*‐*T* deformation conditions or strain rate along the fault or (b) their local obliteration due to pervasive fluid‐rock interaction and cataclasis.

Indeed, hydrothermal alteration was pervasive during brittle faulting as recorded by chloritization of amphibole and biotite, and albitization/saussuritization of plagioclase. This greenschist‐to sub‐greenschist‐facies alteration indicates temperatures of 250–350°C (Gomila et al., 2021) as well as the stable mineral assemblage of the green cataclasites, including chlorite + epidote + albite + quartz (Figures 10 and Tables 1 and 2), consistent with the observations from the Caleta Coloso Fault Zone (Arancibia et al., 2014).

The widespread occurrence of pseudotachylytes documents the ancient seismicity of the BFZ as well as of the strike‐slip NW‐striking faults cutting the CCSZ (Figures 3, 7, 9b–9d, and 10f and 10g). Pseudotachylytes are either pristine or strongly altered and spatially associated with epidote‐chlorite‐bearing veins (Figures 9e–9h). This indicates that seismic faulting occurred in presence of fluids (Gomila et al., 2021). Brittle faulting along the AFS developed once the magmatism waned (Scheuber & Andriessen, 1990; Scheuber & González, 1999). However, the geothermal gradient remained elevated (∼50°C/km) within the abandoned magmatic arc till ∼100 Ma as documented by the cooling evolution of plutons along the El Salado segment (Seymour et al., 2020). Thus, such elevated geothermal gradient rose the brittle‐ductile transition at 5–7 km depth (Arancibia et al., 2014; Cembrano et al., 2005; Seymour et al., 2020). As a result, we infer that the ambient conditions for seismogenic faulting were ≤ 300°C and 5–7 km depth in a fluid‐rich environment (Figures 11d).

#### 6.1.3 Stage 5: Post‐Bolfin Fault Zone s.s. (*T* < 150°C)

The red‐to‐purple‐colored fault gouges, and the calcite‐bearing and hematite‐bearing lineated fault surfaces overprint the BFZ s.s (Figures 9a and 9f). These late Cenozoic faults accommodated normal dip‐slip to oblique strike‐slip displacement associated with reactivation of the AFS (Figure 11e). Fault reactivation was associated with a change of the plate convergence from the SE‐directed oblique subduction during Jurassic and Cretaceous to the first NE‐directed and the latter ENE‐directed oblique subduction during Cenozoic (Pardo‐Casas & Molnar, 1987; Scheuber & González, 1999). Moreover, the Coastal Cordillera became part of the forearc of the Central Andes since Cenozoic. Brittle faulting has been related to co‐seismic to post‐seismic quasi‐elastic rebound in the upper plate due to megathrust earthquakes along the Chile‐Peru trench (e.g., González et al., 2003, 2006), associated with the ENE‐trending subduction of the Nazca oceanic plate (Veloso et al., 2015, and references therein). The fault mineral assemblage, including calcite + palygorskite + halite + gypsum + hematite, indicates temperatures < 150°C (e.g., Bradbury et al., 2015; Morton et al., 2012). The local occurrence of type II twins in calcite grains and clasts within veins suggests however that the deformation temperatures were locally ≥200°C (Ferrill et al., 2004). Cenozoic faulting occurred at shallow crustal levels (<2–3 km depth), consistently with the stratigraphic constraints, as indicated by (a) well‐developed S‐C foliation within fault gouges associated with plastic deformation of gypsum (Figure 10h) (b) and low‐temperature twinning of calcite within the veins (Ferrill et al., 2004).

### 6.2 Role of Precursory Structures on Nucleation of Large‐Scale Seismogenic Faults

The BFZ has a sinuous fault trace and, although being mostly sub‐vertical, the BFZ dip changes from SW to W (northern segment: Playa Escondida and Sand Quarry localities) and NE (southern segment: Quebrada Larga locality) (Figure 3). This change in dip depends on the control on the BFZ orientation by precursory anisotropies as observed for several mesoscale faults hosted in crystalline basement rocks elsewhere (e.g., d'Alessio & Martel, 2005; Di Toro & Pennacchioni, 2005; Griffith et al., 2008). Indeed, the BFZ exploited the magmatic foliation of the Bolfin Complex and the Cerro Cristales Pluton along its northern (Playa Escondida and Sand Quarry localities) and central segments (Quebrada Corta and Ni Miedo localities) (Figures 3, 6, and 8). The NW‐striking subsidiary cataclasites and associated pseudotachylytes within the damage zone nucleated on NE‐dipping andesitic dykes within the isotropic tonalites and granodiorites of the Cerro Cristales Pluton along the southernmost segment (Quebrada Larga locality) (Figures 3 and 9d).

**Figure 12:**
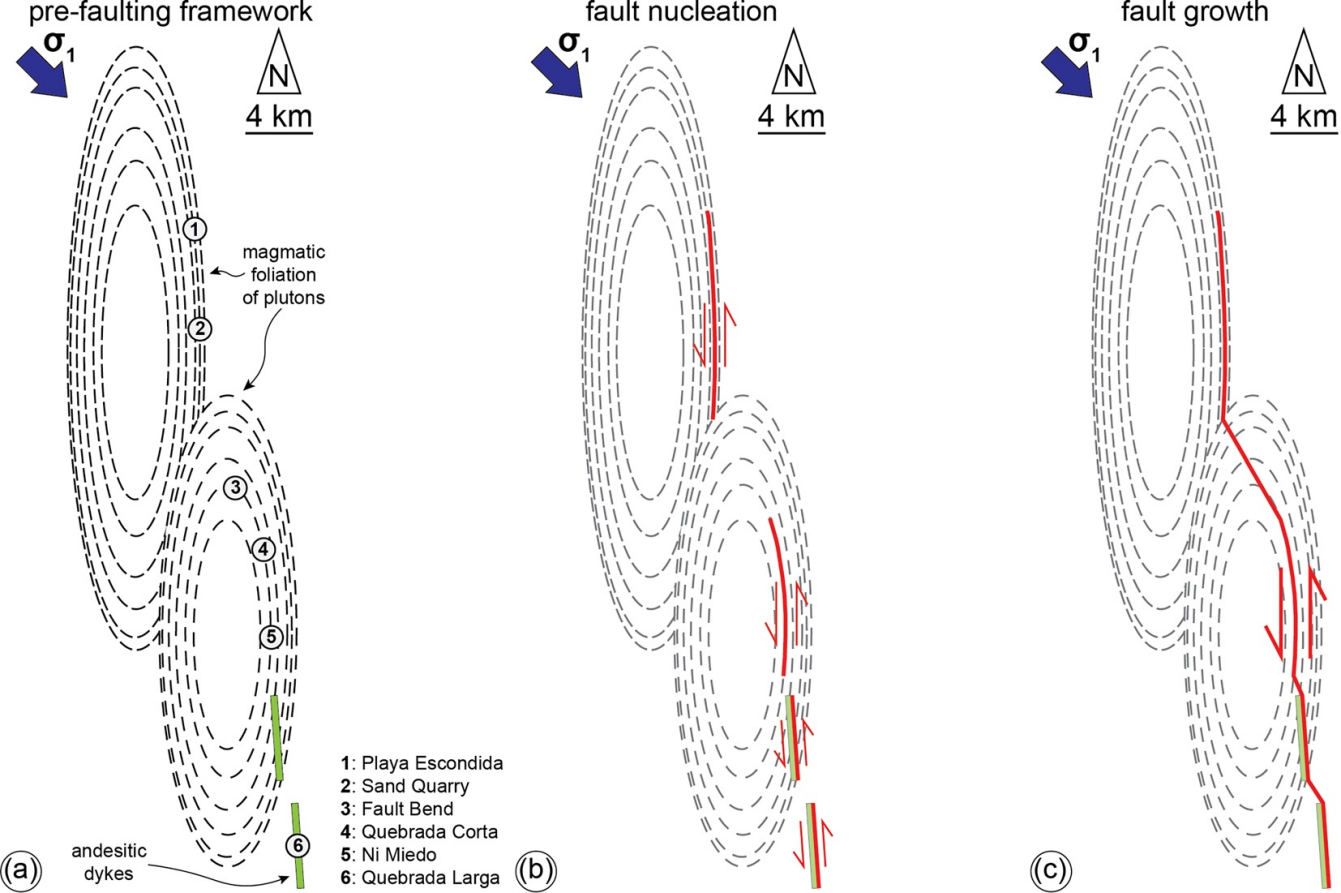
Conceptual model of the evolution of the Bolfin Fault Zone. (a) Pre‐faulting structural framework. (b) Nucleation of the early BFZ segments along structures favorably oriented with respect to the inferred far‐stress field associated with the ancient oblique subduction. The precursory geometrical anisotropies exploited by the brittle faults include the magmatic foliation of plutons (northern and central segments) and andesitic dyke swarm (southern segment). This produces overstepping, N‐to‐NNW‐striking, sinistral strike‐slip fault segments. (c) Fault growth: NW‐striking splay faults developed at the tip of the anisotropy‐pinned fault segments. The progressive linkage of the fault segments resulted in the sinuous geometry of the BFZ.

The reactivation of a precursory structure is controlled by its orientation with respect to the local stress field. In the Bolfin area, the brittle faults of the AFS are organized in strike‐slip duplexes, which partitioned deformation into hierarchically arranged faults, and the BFZ is a third‐order fault splaying out from the second‐order Caleta Coloso Fault Zone (Cembrano et al., 2005). Thus, in the framework of sinistral intra‐arc deformation imposed by the ancient subduction of the Aluk (Phoenix) plate, that is, NW‐SE sub‐horizontal *σ*_1_ (Figures 11d) (Brown et al., 1993; Cembrano et al., 2005; Scheuber & González, 1999; Veloso et al., 2015), the optimal direction for third‐order splay faults to accommodate sinistral strike‐slip shearing should be ∼NNW‐SSE. As a result, the magmatic foliation of the foliated meta‐diorites, tonalites, and granodiorites was optimally oriented to be reactivated as a sinistral strike‐slip fault in the Early Cretaceous tectonic framework (Figure 12a). Instead, the NE‐dipping andesitic dykes were favorably oriented for NNW‐striking sinistral strike‐slip fault reactivation related to a NW‐SE sub‐horizontal *σ*_1_. Several studies however pointed out that faults interaction perturb the regional stress field at fault tip and linkage causing a local stress reorientation (e.g., d'Alessio & Martel, 2004; Kim et al., 2003, 2004; Pachell & Evans, 2002; Segall & Pollard, 1983). Thus, the exploitation of the andesitic dykes may also be partly related to local stress reorientation induced by the interaction between the southernmost proto‐segment of the BFZ and the central proto‐segment of the Caleta Coloso Fault Zone (Figure 12b). On the contrary, where misoriented, the precursory structures are cut by the BFZ, which, for instance, displace the CCSZ of 1 km (between Quebrada Museo and CCSZ‐North localities).

We propose that the nucleation of the BFZ occurred through the exploitation of favorably oriented precursory geometrical anisotropies (i.e., magmatic foliations and dykes). Thus, the BFZ formed as a series of overstepping anisotropy‐pinned fault segments (Figure 12b). During fault growth, NW‐striking splay and horsetail linkage faults developed at the tip of these fault segments (e.g., Fault Bend locality) (Figure 12c). The progressive growth of the BFZ occurred through hard linkages of anisotropy‐pinned fault segments related to the precursory evolution of the magmatic arc and explained the complex and sinuous geometry of the BFZ (Figure 12).

Based on this model, we propose that magmatic‐related structures, such as foliated plutons whose magmatic foliation can extend for several kilometers and dyke swarms, play a pivotal role in controlling the geometry of crustal‐scale faults within magmatic arcs, as do cooling joints at the scale of mesoscale faults within a single pluton (e.g., Di Toro & Pennacchioni, 2005; Pennacchioni et al., 2006; Segall & Pollard, 1983; Smith et al., 2013). Indeed, the exploitation of km‐long foliated plutons and dyke swarms (fault nucleation stage) and consequent linkage of anisotropy‐controlled segments (fault growth stage) could lead to the formation of non‐planar faults with either sinuous trace, as the case of the BFZ, and concave‐shaped trace, such as the first‐order faults of the AFS. The latter was partially documented along the El Salado segment (Figure 1a), where the main fault branch exploited the mylonitic foliation of syn‐magmatic thermal aureoles bounding several Late Jurassic to Early Cretaceous plutons (Brown et al., 1993; Espinoza et al., 2014; Grocott & Taylor, 2002; Seymour et al., 2020). Fault localization along these anisotropies might be promoted by the syn‐kinematic emplacement of both the Late Jurassic‐Early Cretaceous plutons, which are ∼N‐S‐elongated, and dyke swarms, controlled by the same far‐stress field associated with brittle faulting along the AFS.

## 7 Conclusions

We described the spatial and temporal distribution of dykes, magmatic and solid‐state foliations, and brittle faults along the seismogenic BFZ and the syn‐to post‐magmatic CCSZ in the Coastal Cordillera in northern Chile (Figures 1, 2, 3). By combining field geologic surveys, analysis of satellite and drone images, and microstructural and microanalytical observations, we reconstructed the spatio‐temporal evolution of the BFZ, a >40‐km‐long seismogenic splay fault of the 1000‐km‐long strike‐slip AFS. The structural evolution of the BFZ includes five main deformation stages (Figure 11):

- *Stage 1:* diachronous magmatic intrusions of the Bolfin Complex and of the Cerro Cristales Pluton, and formation of magmatic foliations and of large‐scale syn‐to post‐magmatic shear zones. The CCSZ formed at *T* > 700°C and at < 10 km depth during pluton emplacement (Figures 11a);
- *Stage 2:* early post‐magmatic emplacement of leucocratic dykes. Leucocratic and amphibolitic dykes were exploited as strike‐slip ductile shear zones active at >700°C (Figures 11b);
- *Stage 3:* emplacement of multiple generations of dykes arranged into two sub‐vertical sets striking NW and NE, which mutually cut each other (Figures 11c);
- *Stage 4:* formation of the BFZ s.s. The ancient seismogenic behavior is attested by the occurrence of pseudotachylytes. Seismic faulting occurred at ≤ 300°C and 5–7 km depth in a fluid‐rich environment (Figures 11d);
- *Stage 5:* extensional post‐Oligocene fault reactivation of the BFZ occurred at < 150°C and shallow crustal levels (<2–3 km depth) (Figures 11e)

The crustal‐scale BFZ has a sinuous geometry, which is controlled by precursory geometrical anisotropies represented by magmatic foliation of plutons (northern and central segments) and dyke swarms (southern segments) (Figure 3). These precursory structures were favorably oriented to be reactivated with respect to the inferred long‐term stress field associated with the ancient oblique subduction. We propose a conceptual model of fault growth including (i) the exploitation of these favorably oriented precursory anisotropies during fault nucleation and (ii) hard linkage of these anisotropy‐pinned fault segments during fault growth, leading to the formation of the sinuous geometry of the BFZ (Figure 12). The fault evolution proposed for the BFZ may be possibly extended to the formation of the AFS and applied to other crustal‐scale faults in the crystalline basement associated with widespread magmatism.

## Data Availability

All the structural data are available at the repository with the following link: http://researchdata.cab.unipd.it/id/eprint/443.

## References

[tect21587-bib-0001] Acocella, V., Bellier, O., Sandri, L., Sébrier, M., & Pramumijoyo, S. (2018). Weak tectono‐magmatic relationships along an obliquely convergent plate boundary: Sumatra, Indonesia. Frontiers in Earth Science, 6. 10.3389/feart.2018.00003

[tect21587-bib-0002] Allmendinger, R. W., Cardozo, N., & Fisher, D. M. (2011). Structural geology algorithms: Vectors & tensors. Cambridge University Press.

[tect21587-bib-0003] Anderson, E. M. (1951). Dynamics of faulting and dyke formations with application to Britain. Oliver & Boy.

[tect21587-bib-0004] Arabasz, W. J. J. (1971). Geological and geophysical studies of the Atacama fault zone in northern Chile (PhD thesis). California Institute of Technology.

[tect21587-bib-0005] Arancibia, G., Fujita, K., Hoshino, K., Mitchell, T. M., Cembrano, J., Gomila, R., et al. (2014). Hydrothermal alteration in an exhumed crustal fault zone: Testing geochemical mobility in the Caleta Coloso Fault, Atacama Fault System, Northern Chile. Tectonophysics, 623, 147–168. 10.1016/j.tecto.2014.03.024

[tect21587-bib-0006] Balázs, A., Matenco, L., Vogt, K., Cloetingh, S., & Gerya, T. (2018). Extensional polarity change in continental rifts: Inferences from 3‐D numerical modeling and observations. Journal of Geophysical Research: Solid Earth, 123(9), 8073–8094. 10.1029/2018JB015643

[tect21587-bib-0007] Becker, T. W., Hashima, A., Freed, A. M., & Sato, H. (2018). Stress change before and after the 2011 M9 Tohoku‐oki earthquake. Earth and Planetary Science Letters, 504, 174–184. 10.1016/j.epsl.2018.09.035

[tect21587-bib-0008] Bellahsen, N., & Daniel, J. M. (2005). Fault reactivation control on normal fault growth: An experimental study. Journal of Structural Geology, 27(4), 769–780. 10.1016/j.jsg.2004.12.003

[tect21587-bib-0009] Bistacchi, A., Massironi, M., & Menegon, L. (2010). Three‐dimensional characterization of a crustal‐scale fault zone: The Pusteria and Sprechenstein fault system (Eastern Alps). Journal of Structural Geology, 32(12), 2022–2041. 10.1016/j.jsg.2010.06.003

[tect21587-bib-0010] Bistacchi, A., Massironi, M., Menegon, L., Bolognesi, F., & Donghi, V. (2012). On the nucleation of non‐Andersonian faults along phyllosilicate‐rich mylonite belts. Geological Society, London, Special Publications, 367(1), 185–199. 10.1144/SP367.13

[tect21587-bib-0011] Bradbury, K. K., Davis, C. R., Shervais, J. W., Janecke, S. U., & Evans, J. P. (2015). Composition, alteration, and texture of fault‐related rocks from SAFOD core and surface outcrop analogs: Evidence for deformation processes and fluid‐rock interactions. Pure and Applied Geophysics, 172(5), 1053–1078. 10.1007/s00024-014-0896-6

[tect21587-bib-0012] Brown, M., Diàz, F., & Grocott, J. (1993). Displacement history of the Atacama Fault System 25°00’S‐27°00’S, northern Chile. The Geological Society of America Bulletin, 102, 1165–1174. 10.1130/0016-7606(1993)105<1165:dhotaf>2.3.co;2

[tect21587-bib-0013] Butler, R. W. H., Bond, C. E., Shipton, Z. K., Jones, R. R., & Casey, M. (2008). Fabric anisotropy controls faulting in the continental crust. Journal of the Geological Society, 165(2), 449–452. 10.1144/0016-76492007-129

[tect21587-bib-0014] Cao, W., Kaus, B. J. P., & Paterson, S. (2016). Intrusion of granitic magma into the continental crust facilitated by magma pulsing and dike‐diapir interactions: Numerical simulations. Tectonics, 35(6), 1575–1594. 10.1002/2015TC004076

[tect21587-bib-0015] Cardozo, N., & Allmendinger, R. W. (2013). Spherical projections with OSXStereonet. Computers & Geosciences, 51, 193–205. 10.1016/j.cageo.2012.07.021

[tect21587-bib-0016] Cembrano, J., González, G., Arancibia, G., Ahumada, I., Olivares, V., & Herrera, V. (2005). Fault zone development and strain partitioning in an extensional strike‐slip duplex: A case study from the Mesozoic Atacama Fault System, Northern Chile. Tectonophysics, 400(1–4), 105–125. 10.1016/j.tecto.2005.02.012

[tect21587-bib-0017] Chemenda, A. I., Cavalié, O., Vergnolle, M., Bouissou, S., & Delouis, B. (2016). Numerical model of formation of a 3‐D strike‐slip fault system. Comptes Rendus Geoscience, 348(1), 61–69. 10.1016/j.crte.2015.09.008

[tect21587-bib-0018] Christiansen, P. P., & Pollard, D. D. (1997). Nucleation, growth and structural development of mylonitic shear zones in granitic rock. Journal of Structural Geology, 19(9), 1159–1172. 10.1016/S0191-8141(97)00025-4

[tect21587-bib-0019] Clemens, J. D. (1998). Observations on the origins and ascent mechanisms of granitic magmas. Journal of the Geological Society, 155, 843–851. 10.1144/gsjgs.155.5.0843

[tect21587-bib-0020] Collanega, L., Siuda, K., Jackson, A.‐L. C., Bell, R. E., Coleman, A. J., Lenhart, A., Breda, A., & Breda, A. (2019). Normal fault growth influenced by basement fabrics: The importance of preferential nucleation from pre‐existing structures. Basin Research, 31(4), 659–687. 10.1111/bre.12327

[tect21587-bib-0021] Cowan, D. S. (1999). Do faults preserve a record of seismic slip? A field geologist's opinion. Journal of Structural Geology, 21(8–9), 995–1001. 10.1016/S0191-8141(99)00046-2

[tect21587-bib-0022] Crider, J. G. (2015). The initiation of brittle faults in crystalline rock. Journal of Structural Geology, 77, 159–174. 10.1016/j.jsg.2015.05.001

[tect21587-bib-0023] Crider, J. G., & Peacock, D. C. P. (2004). Initiation of brittle faults in the upper crust: A review of field observations. Journal of Structural Geology, 26(4), 691–707. 10.1016/j.jsg.2003.07.007

[tect21587-bib-0025] d'Alessio, M. A., & Martel, S. J. (2004). Fault terminations and barriers to fault growth. Journal of Structural Geology, 26(10), 1885–1896. 10.1016/j.jsg.2004.01.010

[tect21587-bib-0024] d'Alessio, M., & Martel, S. J. (2005). Development of strike‐slip faults from dikes, Sequoia National Park, California. Journal of Structural Geology, 27(1), 35–49. 10.1016/j.jsg.2004.06.013

[tect21587-bib-0026] Davatzes, N. C., & Aydin, A. (2003). The formation of conjugate normal fault systems in folded sandstone by sequential jointing and shearing, Waterpocket monocline, Utah. Journal of Geophysical Research: Solid Earth, 108(B10). 10.1029/2002JB002289

[tect21587-bib-0028] Di Toro, G., & Pennacchioni, G. (2005). Fault plane processes and mesoscopic structure of a strong‐type seismogenic fault in tonalites (Adamello batholith, Southern Alps). Tectonophysics, 402(1–4), 55–80. 10.1016/j.tecto.2004.12.036

[tect21587-bib-0029] Di Toro, G., Pennacchioni, G., & Nielsen, S. (2009). Pseudotachylytes and earthquake source mechanics. Fault‐zone properties and earthquake rupture dynamics (pp. 87–133). 10.1016/S0074-6142(08)00005-3

[tect21587-bib-0030] Domagala, J. P., Escribano, J., De La Cruz, R., Saldias, J., & Joquera, R. (2016). Cartas Blanco Encalada y Pampa Remiendos, Region de Antofagasta. Servicio Nacional de Geología y Minería, Carta Geológica de Chile, Serie Geología Básica 187‐188 mapa escala 1:100.000. Santiago.

[tect21587-bib-0031] Dunai, T. J., González López, G. A., & Juez‐Larré, J. (2005). Oligocene–Miocene age of aridity in the Atacama Desert revealed by exposure dating of erosion‐sensitive landforms. Geology, 33(4), 321. 10.1130/G21184.1

[tect21587-bib-0032] Espinoza, M., Contreras, J. P., Jorquera, R., De La Cruz, R., Kraus, S., Ramirez, C., & Naranjo, J. (2014). Carta Cerro del Pingo, Regiones de Antofagasta y Atacama. Servicio Nacional de Geología y Minería, Carta Geológica de Chile, Serie Geología Básica 169, 1 mapa escala 1:100.000. Santiago.

[tect21587-bib-0033] Ferrill, D. A., Morris, A. P., Evans, M. A., Burkhard, M., Groshong, R. H., & Onasch, C. M. (2004). Calcite twin morphology: A low‐temperature deformation geothermometer. Journal of Structural Geology, 26(8), 1521–1529. 10.1016/j.jsg.2003.11.028

[tect21587-bib-0034] Fondriest, M., Balsamo, F., Bistacchi, A., Clemenzi, L., Demurtas, M., Storti, F., & Di Toro, G. (2020). Structural complexity and mechanics of a shallow crustal seismogenic source (Vado di Corno Fault Zone, Italy). Journal of Geophysical Research: Solid Earth, 125(9). 10.1029/2019JB018926

[tect21587-bib-0035] Fondriest, M., Smith, S. A. F., Di Toro, G., Zampieri, D., & Mittempergher, S. (2012). Fault zone structure and seismic slip localization in dolostones, an example from the Southern Alps, Italy. Journal of Structural Geology, 45, 52–67. 10.1016/j.jsg.2012.06.014

[tect21587-bib-0036] Gomila, R., Arancibia, G., Mitchell, T. M., Cembrano, J. M., & Faulkner, D. R. (2016). Palaeopermeability structure within fault‐damage zones: A snap‐shot from microfracture analyses in a strike‐slip system. Journal of Structural Geology, 83, 103–120. 10.1016/j.jsg.2015.12.002

[tect21587-bib-0126] Gomila, R., Fondriest, M., Jensen, E., Spagnuolo, E., Masoch, S., Mitchell, T. M., et al. (2021). Frictional Melting in Hydrothermal Fluid‐Rich Faults: Field and Experimental Evidence From the Bolfín Fault Zone (Chile). Geochemistry, Geophysics, Geosystems, 22(7). 10.1029/2021gc009743 PMC8365670 34434077

[tect21587-bib-0037] González, G. (1999). Mecanismo y profundidad de emplazamiento del Pluton de Cerro Cristales, Cordillera de la Costa, Antofagasta, Chile. Revista Geologica de Chile, 26(1), 43–66. 10.4067/s0716-02081999000100003

[tect21587-bib-0038] González, G., Cembrano, J., Carrizo, D., Macci, A., & Schneider, H. (2003). The link between forearc tectonics and Pliocene‐Quaternary deformation of the Coastal Cordillera, northern Chile. Journal of South American Earth Sciences, 16(5), 321–342. 10.1016/S0895-9811(03)00100-7

[tect21587-bib-0039] González, G., Dunai, T., Carrizo, D., & Allmendinger, R. (2006). Young displacements on the Atacama Fault System, northern Chile from field observations and cosmogenic 21Ne concentrations. Tectonics, 25(3), 1–n. 10.1029/2005TC001846

[tect21587-bib-0040] González, G., & Niemeyer, H. (2005). Cartas Antofagasta y Punta Tetas, Region de Antofagasta. Servicio Nacional de Geología y Minería, Carta Geológica de Chile, Serie Geología Básica 89 mapa escala 1:100.000. Santiago.

[tect21587-bib-0041] Griffith, W. A., Di Toro, G., Pennacchioni, G., & Pollard, D. D. (2008). Thin pseudotachylytes in faults of the Mt. Abbot quadrangle, Sierra Nevada: Physical constraints for small seismic slip events. Journal of Structural Geology, 30(9), 1086–1094. 10.1016/j.jsg.2008.05.003

[tect21587-bib-0042] Grocott, J., Brown, M., Dallmeyer, R. D., Taylor, G. K., & Treloar, P. J. (1994). Mechanisms of continental growth in extensional arcs: An example from the Andean plate‐boundary zone. Geology, 22, 391–394. 10.1130/0091-7613(1994)022<0391:mocgie>2.3.co;2

[tect21587-bib-0043] Grocott, J., & Taylor, G. K. (2002). Magmatic arc fault systems, deformation partitioning and emplacement of granitic complexes in the Coastal Cordillera, north Chilean Andes (25°30’S to 27°00’S). Journal of the Geological Society, 159(4), 425–443. 10.1144/0016-764901-124

[tect21587-bib-0044] Hardebeck, J. L., & Okada, T. (2018). Temporal stress changes caused by earthquakes: A review. Journal of Geophysical Research: Solid Earth, 123(2), 1350–1365. 10.1002/2017JB014617

[tect21587-bib-0045] Herrera, V., Cembrano, J., Olivares, V., Kojima, S., & Arancibia, G. (2005). Precipitación por despresurización y ebullición en vetas hospedadas en un dúplex de rumbo extensional: Evidencias microestructurales y microtermométricas. Revista Geologica de Chile, 32(2), 207–227. 10.4067/s0716-02082005000200003

[tect21587-bib-0046] Hervé, F., Faundez, V., Calderón, M., Massonne, H.‐J., & Willner, A. P. (2007). Metamorphic and plutonic basement complexes. In T. Moreno, & W. Gibbons (Eds.), The geology of Chile (pp. 5–19). 10.1144/GOCH.2

[tect21587-bib-0047] Hodge, M., Fagereng, Å., Biggs, J., & Mdala, H. (2018). Controls on early‐rift geometry: New perspectives from the Bilila‐Mtakataka Fault, Malawi. Geophysical Research Letters, 45(9), 3896–3905. 10.1029/2018GL077343

[tect21587-bib-0048] Holdsworth, R. E., van Diggelen, E. W. E., Spiers, C. J., de Bresser, J. H. P., Walker, R. J., & Bowen, L. (2011). Fault rocks from the SAFOD core samples: Implications for weakening at shallow depths along the San Andreas Fault, California. Journal of Structural Geology, 33(2), 132–144. 10.1016/j.jsg.2010.11.010

[tect21587-bib-0049] Holland, T., & Blundy, J. (1994). Non‐ideal interactions in calcic amphiboles and their bearing on amphibole‐plagioclase thermometry. Contributions to Mineralogy and Petrology, 116(4), 433–447. 10.1007/BF00310910

[tect21587-bib-0050] Jaeger, J. C., Cook, N. G. W., & Zimmerman, R. (2009). Fundamentals of rock mechanics (4th ed.). Wiley‐Blackwell.

[tect21587-bib-0051] Jaillard, E., Soler, P., Carlier, G., & Mourier, T. (1990). Geodynamic evolution of the northern and central Andes during early to middle Mesozoic times: A Tethyan model. Journal of the Geological Society, 147(6), 1009–1022. 10.1144/gsjgs.147.6.1009

[tect21587-bib-0052] Jensen, E., Cembrano, J., Faulkner, D., Veloso, E., & Arancibia, G. (2011). Development of a self‐similar strike‐slip duplex system in the Atacama Fault system, Chile. Journal of Structural Geology, 33(11), 1611–1626. 10.1016/j.jsg.2011.09.002

[tect21587-bib-0053] Kim, Y. S., Peacock, D. C. P., & Sanderson, D. J. (2003). Mesoscale strike‐slip faults and damage zones at Marsalforn, Gozo Island, Malta. Journal of Structural Geology, 25(5), 793–812. 10.1016/S0191-8141(02)00200-6

[tect21587-bib-0054] Kim, Y. S., Peacock, D. C. P., & Sanderson, D. J. (2004). Fault damage zones. Journal of Structural Geology, 26(3), 503–517. 10.1016/j.jsg.2003.08.002

[tect21587-bib-0055] Kirkpatrick, J. D., Bezerra, F. H. R., Shipton, Z. K., Do Nascimento, A. F., Pytharouli, S. I., Lunn, R. J., & Soden, A. M. (2013). Scale‐dependent influence of pre‐existing basement shear zones on rift faulting: A case study from NE Brazil. Journal of the Geological Society, 170(2), 237–247. 10.1144/jgs2012-043

[tect21587-bib-0056] Lara, L. E., Naranjo, J. A., & Moreno, H. (2004). Rhyodacitic fissure eruption in Southern Andes (Cordón Caulle; 40.5°S) after the 1960 (Mw: 9.5) Chilean earthquake: A structural interpretation. Journal of Volcanology and Geothermal Research, 138(1–2), 127–138. 10.1016/j.jvolgeores.2004.06.009

[tect21587-bib-0057] Lucassen, F., & Franz, G. (1994). Arc related Jurassic igneous and meta‐igneous rocks in the Coastal Cordillera of northern Chile/Region Antofagasta. Lithos, 32(3–4), 273–298. 10.1016/0024-4937(94)90044-2

[tect21587-bib-0058] Lucassen, F., & Thirlwall, M. F. (1998). Sm–Nd ages of mafic rocks from the Coastal Cordillera at 24°S , northern Chile. Geologische Rundschau, 86, 767–774. 10.1007/s005310050175

[tect21587-bib-0059] Lupi, M., & Miller, S. A. (2014). Short‐lived tectonic switch mechanism for long‐term pulses of volcanic activity after mega‐thrust earthquakes. Solid Earth, 5(1), 13–24. 10.5194/se-5-13-2014

[tect21587-bib-0060] Lupi, M., Trippanera, D., Gonzalez, D., D'amico, S., Acocella, V., Cabello, C., Tassara, A., & Tassara, A. (2020). Transient tectonic regimes imposed by megathrust earthquakes and the growth of NW‐trending volcanic systems in the Southern Andes. Tectonophysics, 774, 228204. 10.1016/j.tecto.2019.228204

[tect21587-bib-0061] Mancktelow, N., & Pennacchioni, G. (2020). Intermittent fracturing in the middle continental crust as evidence for transient switching of principal stress axes associated with the subduction zone earthquake cycle. Geology, 48, 1072–1076. 10.1130/G47625.1

[tect21587-bib-0062] Mandl, G. (1988). Mechanics of tectonic faulting: Models and basic concepts. In H. J. Zwart (Ed.). Elsevier.

[tect21587-bib-0063] Martel, S. J. (1990). Formation of compound strike‐slip fault zones, Mount Abbot quadrangle, California. Journal of Structural Geology, 12(7), 869–882. 10.1016/0191-8141(90)90060-C

[tect21587-bib-0064] Massironi, M., Bistacchi, A., & Menegon, L. (2011). Misoriented faults in exhumed metamorphic complexes: Rule or exception? Earth and Planetary Science Letters, 307(1–2), 233–239. 10.1016/j.epsl.2011.04.041

[tect21587-bib-0065] Miller, R. B., & Paterson, S. R. (1999). In defense of magmatic diapirs. Journal of Structural Geology, 21(8–9), 1161–1173. 10.1016/S0191-8141(99)00033-4

[tect21587-bib-0066] Mitchell, T. M., & Faulkner, D. R. (2009). The nature and origin of off‐fault damage surrounding strike‐slip fault zones with a wide range of displacements: A field study from the Atacama Fault System, northern Chile. Journal of Structural Geology, 31(8), 802–816. 10.1016/j.jsg.2009.05.002

[tect21587-bib-0067] Mittempergher, S., Zanchi, A., Zanchetta, S., Fumagalli, M., Gukov, K., & Bistacchi, A. (2021). Fault reactivation and propagation in the northern Adamello pluton: The structure and kinematics of a kilometer‐scale seismogenic source. Tectonophysics, 806, 228790. 10.1016/j.tecto.2021.228790

[tect21587-bib-0068] Molina, J. F., Moreno, J. A., Castro, A., Rodríguez, C., & Fershtater, G. B. (2015). Calcic amphibole thermobarometry in metamorphic and igneous rocks: New calibrations based on plagioclase/amphibole Al‐Si partitioning and amphibole/liquid Mg partitioning. Lithos, 232, 286–305. 10.1016/j.lithos.2015.06.027

[tect21587-bib-0069] Morton, N., Girty, G. H., & Rockwell, T. K. (2012). Fault zone architecture of the San Jacinto fault zone in Horse Canyon, southern California: A model for focused post‐seismic fluid flow and heat transfer in the shallow crust. Earth and Planetary Science Letters, 329(330), 71–83. 10.1016/j.epsl.2012.02.013

[tect21587-bib-0070] Naliboff, J. B., Glerum, A., Brune, S., Péron‐Pinvidic, G., & Wrona, T. (2020). Development of 3‐D rift heterogeneity through fault network evolution. Geophysical Research Letters, 47(13), e2019GL086611. 10.1029/2019GL086611

[tect21587-bib-0071] Nasseri, M. H., Rao, K. S., & Ramamurthy, T. (1997). Failure mechanism in schistose rocks. International Journal of Rock Mechanics and Mining Sciences, 34(3–4), 219.e1–219.e15. 10.1016/S1365-1609(97)00099-3

[tect21587-bib-0072] Nasseri, M. H., Rao, K. S., & Ramamurthy, T. (2003). Anisotropic strength and deformational behavior of Himalayan schists. International Journal of Rock Mechanics and Mining Sciences, 40(1), 3–23. 10.1016/S1365-1609(02)00103-X

[tect21587-bib-0073] Naylor, M., Mandl, G., & Supesteijn, C. H. (1986). Fault geometries in basement‐induced wrench faulting under different initial stress states. Journal of Structural Geology, 8(7), 737–752. 10.1016/0191-8141(86)90022-2

[tect21587-bib-0074] Olivares, V., Herrera, V., Cembrano, J., Arancibia, G., Reyes, N., & Faulkner, D. (2010). Tectonic significance and hydrothermal fluid migration within a strike‐slip duplex fault‐vein network: An example from the Atacama Fault System. Andean Geology, 37, 473–497. 10.5027/andgeov37n2-a12

[tect21587-bib-0075] Pachell, M. A., & Evans, J. P. (2002). Growth, linkage, and termination processes of a 10‐km‐long strike‐slip fault in jointed granite: The Gemini fault zone, Sierra Nevada, California. Journal of Structural Geology, 24(12), 1903–1924. 10.1016/S0191-8141(02)00027-5

[tect21587-bib-0076] Parada, M. A., López‐Escobar, L., Oliveros, V., Fuentes, F., Morata, D., Calderón, M., et al. (2007). Andean magmatism. In T. Moreno, & W. Gibbons (Eds.), The geology of Chile (pp. 115–146). 10.1144/GOCH.4

[tect21587-bib-0077] Pardo‐Casas, F., & Molnar, P. (1987). Relative motion of the Nazca (Farallon) and South American Plates since Late Cretaceous time. Tectonics, 6(3), 233–248. 10.1029/TC006i003p00233

[tect21587-bib-0078] Paterson, S. R., & Vernon, R. H. (1995). Bursting the bubble of ballooning plutons: A return to nested diapirs emplaced by multiple processes. The Geological Society of America Bulletin, 107(11), 1356–1380. 10.1130/0016-7606(1995)107<1356:btbobp>2.3.co;2

[tect21587-bib-0079] Peacock, D. C. P., & Sanderson, D. J. (1995). Strike‐slip relay ramps. Journal of Structural Geology, 17(10), 1351–1360. 10.1016/0191-8141(95)97303-W

[tect21587-bib-0080] Pearce, R. K., Sánchez de la Muela, A., Moorkamp, M., Hammond, J. O. S., Mitchell, T. M., Cembrano, J., et al. (2020). Reactivation of fault systems by compartmentalized hydrothermal fluids in the Southern Andes revealed by magnetotelluric and seismic data. Tectonics, 39. 10.1029/2019TC005997

[tect21587-bib-0081] Pennacchioni, G. (2005). Control of the geometry of precursor brittle structures on the type of ductile shear zone in the Adamello tonalites, Southern Alps (Italy). Journal of Structural Geology, 27(4), 627–644. 10.1016/j.jsg.2004.11.008

[tect21587-bib-0082] Pennacchioni, G., Di Toro, G., Brack, P., Menegon, L., & Villa, I. M. (2006). Brittle‐ductile‐brittle deformation during cooling of tonalite (Adamello, Southern Italian Alps). Tectonophysics, 427(1–4), 171–197. 10.1016/j.tecto.2006.05.019

[tect21587-bib-0083] Pennacchioni, G., & Mancktelow, N. S. (2013). Initiation and growth of strike‐slip faults within intact metagranitoid (Neves area, eastern Alps, Italy). Bulletin of the Geological Society of America, 125(9–10), 1468–1483. 10.1130/B30832.1

[tect21587-bib-0084] Pennacchioni, G., & Mancktelow, N. S. (2018). Small‐scale ductile shear zones: Neither extending, nor thickening, nor narrowing. Earth‐Science Reviews, 184, 1–12. 10.1016/j.earscirev.2018.06.004

[tect21587-bib-0085] Pennacchioni, G., & Zucchi, E. (2013). High temperature fracturing and ductile deformation during cooling of a pluton: The Lake Edison granodiorite (Sierra Nevada batholith, California). Journal of Structural Geology, 50, 54–81. 10.1016/J.JSG.2012.06.001

[tect21587-bib-0086] Pérez‐Flores, P., Cembrano, J., Sánchez‐Alfaro, P., Veloso, E., Arancibia, G., & Roquer, T. (2016). Tectonics, magmatism and paleo‐fluid distribution in a strike‐slip setting: Insights from the northern termination of the Liquiñe–Ofqui fault System, Chile. Tectonophysics, 680, 192–210. 10.1016/j.tecto.2016.05.016

[tect21587-bib-0087] Perrin, C., Manighetti, I., Ampuero, J.‐P., Cappa, F., & Gaudemer, Y. (2016). Location of largest earthquake slip and fast rupture controlled by along‐strike change in fault structural maturity due to fault growth. Journal of Geophysical Research: Solid Earth, 121(5), 3666–3685. 10.1002/2015JB012671

[tect21587-bib-0088] Phillips, T. B., Fazlikhani, H., Gawthorpe, R. L., Fossen, H., Jackson, C. A.‐L., Bell, R. E., et al. (2019). The influence of structural inheritance and multiphase extension on rift development, the Northern North Sea. Tectonics, 38(12), 4099–4126. 10.1029/2019TC005756

[tect21587-bib-0089] Rizza, M., Bollinger, L., Sapkota, S. N., Tapponnier, P., Klinger, Y., Karakaş, Ç., et al. (2019). Post earthquake aggradation processes to hide surface ruptures in thrust systems: The M8.3, 1934, Bihar‐Nepal earthquake ruptures at Charnath Khola (Eastern Nepal). Journal of Geophysical Research: Solid Earth, 124(8), 9182–9207. 10.1029/2018JB016376

[tect21587-bib-0090] Rowe, C. D., & Griffith, W. A. (2015). Do faults preserve a record of seismic slip: A second opinion. Journal of Structural Geology, 78, 1–26. 10.1016/j.jsg.2015.06.006

[tect21587-bib-0091] Ruthven, R., Singleton, J., Seymour, N., Gomila, R., Arancibia, G., Stockli, D. F., et al. (2020). The geometry, kinematics, and timing of deformation along the southern segment of the Paposo fault zone, Atacama Fault System, northern Chile. Journal of South American Earth Sciences, 97, 102355. 10.1016/j.jsames.2019.102355

[tect21587-bib-0092] Sawyer, E. W. (2000). Grain‐scale and outcrop‐scale distribution and movement of melt in a crystallizing granite. Earth and Environmental Science Transactions of the Royal Society of Edinburgh, 91(1–2), 73–85. 10.1017/S0263593300007306

[tect21587-bib-0093] Scheuber, E., & Andriessen, P. A. M. (1990). The kinematic and geodynamic significance of the Atacama fault zone, northern Chile. Journal of Structural Geology, 12(2), 243–257. 10.1016/0191-8141(90)90008-M

[tect21587-bib-0094] Scheuber, E., & González, G. (1999). Tectonics of the Jurassic‐Early Cretaceous magmatic arc of the north Chilean Coastal Cordillera (22°‐26°S): A story of crustal deformation along a convergent plate boundary. Tectonics, 18(5), 895–910. 10.1029/1999TC900024

[tect21587-bib-0095] Scheuber, E., Hammerschmidt, K., & Friedrichsen, H. (1995). 40Ar/39Ar and Rb‐Sr analyses from ductile shear zones from the Atacama Fault Zone, northern Chile: The age of deformation. Tectonophysics, 250(1–3), 61–87. 10.1016/0040-1951(95)00044-8

[tect21587-bib-0096] Scholz, C. H. (2019). The mechanics of earthquakes and faulting. 10.1017/9781316681473

[tect21587-bib-0097] Segall, P., & Pollard, D. P. (1983). Nucleation and growth of strike slip faults in granite. Journal of Geophysical Research, 88(B1), 555–568. 10.1029/JB088iB01p00555

[tect21587-bib-0098] Segall, P., & Simpson, C. (1986). Nucleation of ductile shear zones on dilatant fractures. Geology, 14(1), 56. 10.1130/0091-7613(1986)14<56:NODSZO>2.0.CO;2

[tect21587-bib-0099] SERNAGEOMIN . (2003). Mapa Geológico de Chile: versión digital. Base geológica escala 1:1.000.000. Santiago: Servicio Nacional de Geología y Minería, Publicación Geológica Digital No. 4 (CD‐ROM, versión1.0, 2003).

[tect21587-bib-0100] Seymour, N. M., Singleton, J. S., Gomila, R., Mavor, S. P., Heuser, G., Arancibia, G., et al. (2021). Magnitude, timing, and rate of slip along the Atacama Fault System, northern Chile: Implications for Early Cretaceous slip partitioning and plate convergence. Journal of the Geological Society, 178, jgs2020‐142. 10.1144/jgs2020-142

[tect21587-bib-0101] Seymour, N. M., Singleton, J. S., Mavor, S. P., Gomila, R., Stockli, D. F., Heuser, G., & Arancibia, G. (2020). The relationship between magmatism and deformation along the intra‐arc strike‐slip Atacama fault system, Northern Chile. Tectonics, 39(3), e2019TC005702. 10.1029/2019TC005702

[tect21587-bib-0102] Shigematsu, N., Kametaka, M., Inada, N., Miyawaki, M., Miyakawa, A., Kameda, J., Fujimoto, K., et al. (2017). Evolution of the Median Tectonic Line fault zone, SW Japan, during exhumation. Tectonophysics, 696–697, 52–69. 10.1016/j.tecto.2016.12.017

[tect21587-bib-0103] Sibson, R. H. (1975). Generation of pseudotachylyte by ancient seismic faulting. Geophysical Journal of the Royal Astronomical Society, 43(3), 775–794. 10.1111/j.1365-246X.1975.tb06195.x

[tect21587-bib-0104] Sibson, R. H. (1990). Faulting and fluid flow. In B. E. Nesbitt (Ed.), Fluids in tectonically active regimes of the continental crust. Mineralogical Association of Canada, Short Course on Crustal Fluids, Handbook 18 (pp. 93–132).

[tect21587-bib-0105] Sielfeld, G., Lange, D., & Cembrano, J. (2019). Intra‐arc crustal seismicity: Seismotectonic implications for the Southern Andes Volcanic Zone, Chile. Tectonics, 38(2), 552–578. 10.1029/2018TC004985

[tect21587-bib-0106] Smith, S. A. F., Bistacchi, A., Mitchell, T. M., Mittempergher, S., & Di Toro, G. (2013). The structure of an exhumed intraplate seismogenic fault in crystalline basement. Tectonophysics, 599, 29–44. 10.1016/j.tecto.2013.03.031

[tect21587-bib-0107] Snoke, A. W., Tullis, J., & Todd, V. R. (1998). Fault‐related rocks: A photographic atlas. 10.2307/j.ctt7zvg0k

[tect21587-bib-0108] Stewart, M., Holdsworth, R. E., & Strachan, R. A. (2000). Deformation processes and weakening mechanisms within the frictional–viscous transition zone of major crustal‐scale faults: Insights from the Great Glen Fault Zone, Scotland. Journal of Structural Geology, 22(5), 543–560. 10.1016/S0191-8141(99)00164-9

[tect21587-bib-0109] Stipp, M., Stünitz, H., Heilbronner, R., & Schmid, S. M. (2002). The eastern Tonale fault zone: A ‘natural laboratory’ for crystal plastic deformation of quartz over a temperature range from 250 to 700°C. Journal of Structural Geology, 24(12), 1861–1884. 10.1016/S0191-8141(02)00035-4

[tect21587-bib-0110] Storti, F., Holdsworth, R. E., & Salvini, F. (2003). In F. Storti, R. E. Holdsworth, & F. Salvini (Eds.), Intraplate strike‐slip deformation belts (Vol. 210, pp. 1–14). Geological Society, London, Special Publications. 10.1144/GSL.SP.2003.210.01.01

[tect21587-bib-0111] Swanson, M. T. (1988). Pseudotachylyte‐bearing strike‐slip duplex structures in the Fort Foster Brittle Zone, S. Maine. Journal of Structural Geology, 10(8), 813–828. 10.1016/0191-8141(88)90097-1

[tect21587-bib-0112] Swanson, M. T. (1992). Fault structure, wear mechanisms and rupture processes in pseudotachylyte generation. Tectonophysics, 204(3–4), 223–242. 10.1016/0040-1951(92)90309-T

[tect21587-bib-0113] Swanson, M. T. (1999a). Dextral transpression at the Casco Bay restraining bend, Norumbega fault zone, coastal Maine. Norumbega Fault System of the Northern Appalachians. 10.1130/0-8137-2331-0.85

[tect21587-bib-0114] Swanson, M. T. (1999b). Kinematic indicators for regional dextral shear along the Norumbega fault system in the Casco Bay area, coastal Maine. Norumbega fault system of the Northern Appalachians (pp. 1–24). 10.1130/0-8137-2331-0.1

[tect21587-bib-0115] Swanson, M. T. (2006a). Late Paleozoic strike‐slip faults and related vein arrays of Cape Elizabeth, Maine. Journal of Structural Geology, 28(3), 456–473. 10.1016/j.jsg.2005.12.009

[tect21587-bib-0116] Swanson, M. T. (2006b). Pseudotachylyte‐bearing strike‐slip faults in mylonitic host rocks, Fort Foster Brittle Zone, Kittery, Maine. In R. Abercrombie, A. McGarr, G. Di Toro, & H. Kanamori (Eds.), Earthquakes: Radiated energy and the physics of faulting (pp. 167–179). 10.1029/170GM17

[tect21587-bib-0117] Sylvester, A. G. (1988). Strike‐slip faults. The Geological Society of America Bulletin, 100(11), 1666–1703. 10.1130/0016-7606(1988)100<1666:SSF>2.3.CO;2

[tect21587-bib-0118] Veloso, E. E., Gomila, R., Cembrano, J., González, R., Jensen, E., & Arancibia, G. (2015). Stress fields recorded on large‐scale strike‐slip fault systems: Effects on the tectonic evolution of crustal slivers during oblique subduction. Tectonophysics, 664, 244–255. 10.1016/j.tecto.2015.09.022

[tect21587-bib-0119] Wedmore, L. N. J., Williams, J. N., Biggs, J., Fagereng, Å., Mphepo, F., Dulanya, Z., et al. (2020). Structural inheritance and border fault reactivation during active early‐stage rifting along the Thyolo fault, Malawi. Journal of Structural Geology, 139, 104097. 10.1016/j.jsg.2020.104097

[tect21587-bib-0120] Weinberg, R. F. (2006). Melt segregation structures in granitic plutons. Geology, 34(4), 305. 10.1130/G22406.1

[tect21587-bib-0121] Whipp, P. S., Jackson, C. A.‐L., Gawthorpe, R. L., Dreyer, T., & Quinn, D. (2014). Normal fault array evolution above a reactivated rift fabric; a subsurface example from the northern Horda Platform, Norwegian North Sea. Basin Research, 26(4), 523–549. 10.1111/bre.12050

[tect21587-bib-0122] Whitney, D. L., & Evans, B. W. (2010). Abbreviations for names of rock‐forming minerals. American Mineralogist, 95(1), 185–187. 10.2138/am.2010.3371

[tect21587-bib-0123] Williams, J. N., Toy, V. G., Smith, S. A. F., & Boulton, C. (2017). Fracturing, fluid‐rock interaction and mineralization during the seismic cycle along the Alpine Fault. Journal of Structural Geology, 103, 151–166. 10.1016/j.jsg.2017.09.011

[tect21587-bib-0124] Woodcock, N. H. (1986). The role of strike‐slip fault systems at plate boundaries. Philosophical Transactions of the Royal Society of London ‐ Series A: Mathematical and Physical Sciences, 317(1539), 13–29. 10.1098/rsta.1986.0021

